# Inhibition of ZIP4 reverses epithelial-to-mesenchymal transition and enhances the radiosensitivity in human nasopharyngeal carcinoma cells

**DOI:** 10.1038/s41419-019-1807-7

**Published:** 2019-08-05

**Authors:** Qi Zeng, Yi-min Liu, Jun Liu, Jian Han, Jian-xin Guo, Shun Lu, Xue-mei Huang, Ping Yi, Jin-yi Lang, Peng Zhang, Chun-ting Wang

**Affiliations:** 1 State Key Laboratory of Biotherapy/Collaborative Innovation Center of Biotherapy, West China Hospital, Sichuan University, 610041 Chengdu, China; 2 Department of Radiation Oncology, Sichuan Cancer Hospital & Institute, Sichuan Cancer Center, School of Medicine, University of Electronic Science and Technology of China, Radiation Oncology Key Laboratory of Sichuan Province, 610041 Chengdu, China; 3 Department of Gynaecology and Obstetrics, Institute of Surgery Research, Daping Hospital, Army Medical University (Third Military Medical University), 400042 Chongqing, China; 4 Department of Oncology, Sun Yat-sen Memorial Hospital of Sun Yat-sen University, Guangzhou, China; 5 Department of Otorhinolaryngology, Head and Neck Surgey, West China Hospital, Sichuan University, 610041 Chengdu, China

**Keywords:** Cancer therapy, Oncogenes

## Abstract

ZIP4 is a zinc transporter involved in epithelial cell morphology and migration in various cancers. In the epithelial-to-mesenchymal transition (EMT), epithelial cells transition into mesenchymal cells. The EMT plays a crucial role in invasiveness and metastasis during tumorigenesis. The aim of this study was to investigate the role of ZIP4 in the invasiveness and radiosensitivity of human nasopharyngeal carcinoma (NPC). In this study, results from 99 human patients with NPC showed that ZIP4 expression levels significantly correlated with a higher TN (tumor, lymph node) classification, as well as shorter overall survival (OS), recurrence-free survival (RFS), and distant metastasis-free survival (DMFS). Forced overexpression of ZIP4 promoted the migration and invasion of C666-1 cells through regulation of the EMT process. In contrast, ZIP4 silencing by lentivirus-mediated shRNA inhibited the EMT and metastasis of C666-1 cells in vitro and in vivo. Importantly, protein microarray analyses showed that downregulation of ZIP4 in C666-1 cells resulted in the decreased abundance of phosphoinositide 3-kinase (PI3K) p85 (Tyr607), phosphorylated (p)-Akt (Ser473), phosphorylated (p)-Akt (Thr308), and phosphorylated glycogen synthase kinase 3β (pGSK3β; Ser9). These data suggest that ZIP4 induces the EMT and promotes migration and invasion via the PI3K/Akt signaling pathway in NPC. Moreover, ZIP4 silencing significantly enhanced radiation-induced apoptosis and growth inhibition of human C666-1 cells in vitro and enhanced the antitumor activity of ionizing radiation (IR), leading to tumor growth inhibition in vivo. These results demonstrate that ZIP4 is a novel prognostic factor for malignant NPC progression. More importantly, targeting ZIP4, along with radiotherapy, may be an effective new treatment for NPC.

## Introduction

Nasopharyngeal carcinoma (NPC), derived from the nasopharynx epithelium, is an endemic head and neck malignant disease. NPC is one of the most frequently occurring cancers in Southern China and Southeast Asia^1^. Almost 98% of all NPC cases are associated with latent infection with the Epstein–Barr virus (EBV)^2,3^. EBV infection is an early etiologic event in the evolution of NPC^4^. Although many NPC cell lines do not express the EBV genome after long term passage, C666-1 cells retain the native EBV genome. Thus, the C666-1 cell line is an ideal model for investigating EBV-associated NPC^5^.

The World Health Organization (WHO) has classified NPC into three histological types: keratinizing squamous cell carcinoma (WHO type I), differentiated nonkeratinizing squamous cell carcinoma (type II), and undifferentiated carcinomas (type III)^1^. NPC types II and III are more sensitive to ionizing radiation (IR) in comparison with type I. As NPC types II and III are relatively widespread in Southern China and parts of Southeast Asia^6^, radiotherapy is used extensively to treat NPC patients in these areas. Unfortunately, resistance to radiotherapy is a significant obstacle to effective treatment of NPC patients. The combination of radiotherapy and chemotherapy increases survival, however, reappearance at the local site and the development of metastases occur in 30–40% of NPC patients with advanced stage disease because of radiotherapy resistance^7^. This high rate of disease recurrence underscores the need to elucidate the mechanism underlying radiation resistance in NPC patients.

Zinc, an essential micronutrient, is a cofactor for numerous metalloenzymes and transcription factors^8,9^. Therefore, rapidly dividing cells, including cancer cells, require a relatively large amount of zinc^10^. Zinc is transported into cells via zinc importer (ZIP) proteins and exported via zinc transporter (ZnT) proteins^11^. Dysregulated ZnTs have been implicated in several human cancers^12,13^. For example, ZIP4 is a prognostic marker and molecular target in pancreatic tumors^14^. ZIP4 is overexpressed in most pancreatic tumors, and ZIP4 expression is correlated with pancreatic tumor progression and survival^15,16^. Data from murine models indicate that ZIP4 overexpression promotes tumor growth and metastasis^15–18^. Exosomal ZIP4 is associated with cancer growth and can be utilized as a diagnostic biomarker for pancreatic cancer^19^. Furthermore, silencing of ZIP4 attenuates bone loss in pancreatic cancer patients^20^. In addition, as a novel determinant of tumor invasion, ZIP4 contributes to tumor recurrence after liver transplantation in patients with hepatocellular carcinoma (HCC)^21^. High ZIP4 expression is correlated with a higher glioma grade and decreased overall survival (OS)^22^. In contrast with its role in pancreatic and hepatic cancers, ZIP4 has an inhibitory effect on prostate carcinoma cell proliferation and invasion in vitro^23^.

However, the expression profile and functions of zinc transporters in NPC have not been reported. Therefore, the aim of the present study was to investigate the tumorigenic function of ZIP4 in NPC. We found that high ZIP4 expression was associated with metastasis and poor prognosis in human NPC patients. Next, we showed that elevated ZIP4 expression induced the EMT in C666-1 cells by activating the PI3K/Akt pathway. Moreover, downregulation of ZIP4 expression enhanced the radiosensitivity of C666-1 cells and inhibited metastasis. These findings demonstrate a novel role for ZIP4, which could lead to the development of improved treatments for NPC patients.

## Results

### High ZIP4 expression correlates with metastasis and poor prognosis in patients with NPC

**Fig. 1:**
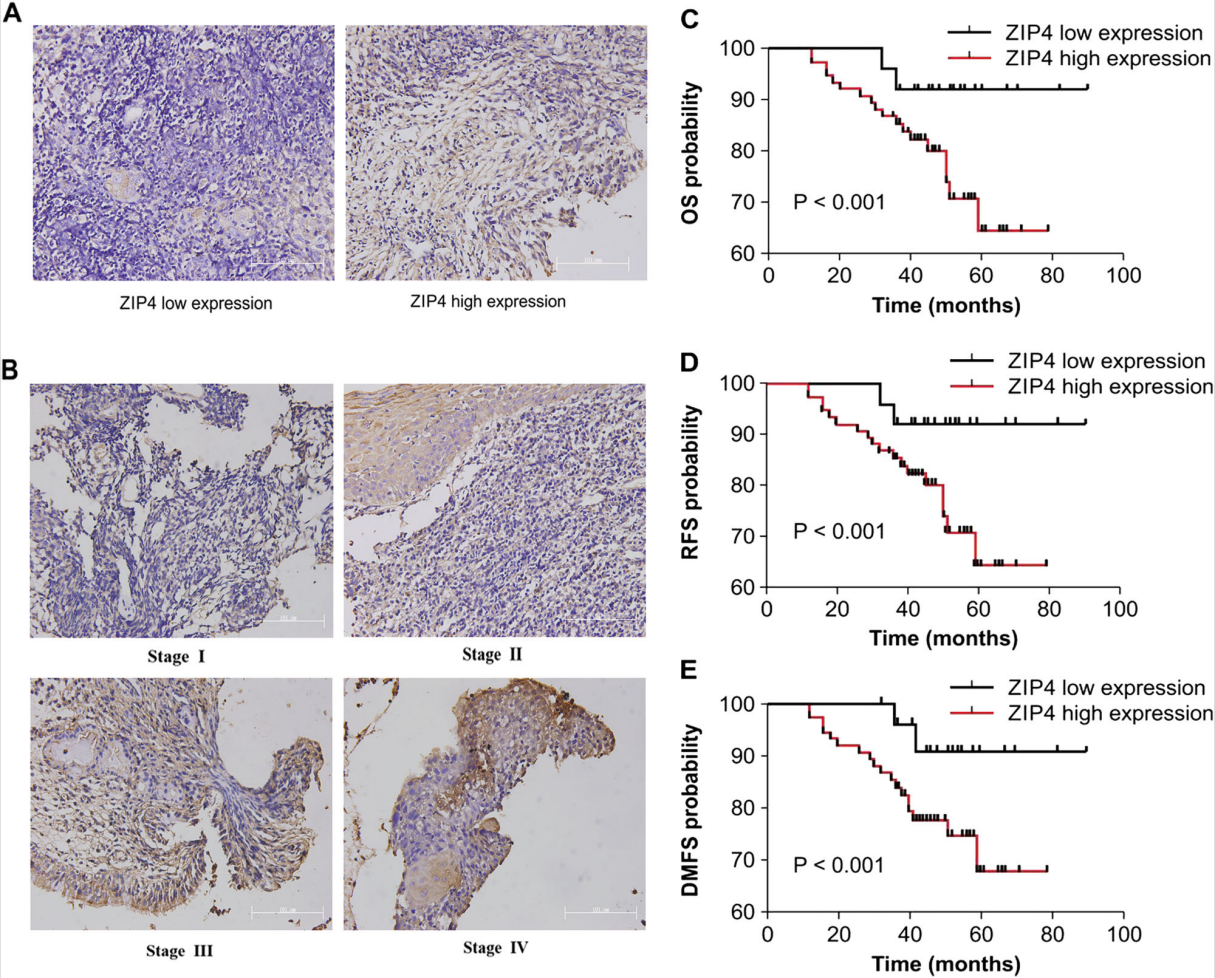
Association of ZIP4 expression with clinicopathological variables in patients with NPC. Representative specimens from patients with NPC showed weak (**a**) and strong (**b**) cell membrane immunostaining for ZIP4 at different NPC stages (Scale bar = 101 μm). The results of the Kaplan–Meier survival analysis indicate that patients with high ZIP4 expression present a shorter overall survival (OS) (**c**), recurrence-free survival (RFS) (**d**), and distant metastasis-free survival (DMFS) (**e**). The differences in OS, RFS, and DMFS between the ZIP4 low and high expression levels were significant, low/high = 25/74 (*P* < 0.001)

Specimens from 99 patients with nonkeratinizing NPC were analyzed using immunohistochemistry (IHC) to determine ZIP4 expression. To clarify the levels of ZIP4 expression, we performed a histological evaluation based on the histoscore (H-score). In ZIP4-positive tissues, positive staining was observed in the tumor cell membrane (Fig. 1a). Based on the principles of invasion area of lesion and progress, the TNM clinical staging system for NPC divides the severity of carcinomas (termed either “in situ” (tumor, T), “regional lymph node metastasis” (node, N) or “distant metastasis” (metastasis, M)) into several levels, such as T_1–4_, N_0–3_, and M_0–1_. Informed by the prognosis of the three abovementioned degrees of severity, the NPC is divided into four clinical stages, from I to IV stages. Overall, in our studies, ZIP4 expression was observed in all patients and was significantly associated with clinical T classification (*P* = 0.0001), N classification (*P* = 0.0017), and clinical stage (*P* = 0.0051) (Fig. 1b). Higher ZIP4 protein expression was observed in patients presented a late stage (T_3–4_, N_2–3_, and III–IV) NPC according to the American Joint Committee on Cancer (AJCC Cancer Staging Manual, 8th edition). No correlation was observed between ZIP4 expression and age or gender (*P* > 0.05) (Table 1).

**Table 1:** Correlation between ZIP4 expression and clinicopathological features in 99 patients with nonkeratinizing NPC

Factors	Number	Average score	*P*-value
Gender			0.0956
Male	77	6.605 ± 0.2858	
Female	22	5.389 ± 0.8407	
Age (years)			0.6731
<60	81	6.333 ± 0.3120	
≥60	18	5.389 ± 0.8407	
T classification			0.0001
T_1_–T_2_	38	4.897 ± 0.4585	
T_3_–T_4_	61	7.102 ± 0.3168	
N classification			0.0017
N_0_–N_1_	27	3.000 ± 0.8452	
N_2_–N_3_	72	8.000 ± 1.0000	
Clinical stage			0.0051
Early (I–II)	12	4.385 ± 1.135	
Late (III–IV)	87	6.686 ± 0.2632	
ZIP4 expression			–
Low	25	–	
High	74	–	

Clinical and TNM staging were referenced to the American Joint Committee on Cancer: AJCC Cancer Staging Manual, 8th edition

The Kaplan–Meier analysis method and the log-rank test were used to relate ZIP4 expression to OS, recurrence-free survival (RFS), and distant metastasis-free survival (DMFS). High ZIP4 protein expression was strongly correlated with worse OS in NPC patients (Fig. 1c, *P* < 0.001). Furthermore, patients with tumors expressing high ZIP4 protein levels had significantly more frequent recurrence or metastasis within 5 years in comparison with patients with low ZIP4 expression (Fig. 1d, e; *P* < 0.001). Using univariate analysis of all patients in the study, clinical stage (hazard ratio [HR] = 1.055, *P* = 0.031) and ZIP4 overexpression (HR = 2.185, *P* < 0.01) were significantly associated with poor OS. More importantly, clinical stage (HR = 1.538, *P* = 0.027) and ZIP4 overexpression (HR = 2.315, *P* = 0.012) were independent predictors for OS based on the multivariate analysis (Table 2).

**Table 2:** Summary of univariate and multivariate analysis of overall survival in all NPCs

Parameters	Univariate analysis			Multivariate analysis		
	HR	95% CI	*P*	HR	95% CI	*P*
Gender						
Male vs. female	1.417	0.689–1.417	0.343			
Age (years)						
<60 vs. ≥60	1.61	0.796–3.256	0.185			
T classification						
T_1_–T_2_ vs. T_3_–T_4_	1.367	0.749–2.496	0.139			
N classification						
N_0_–N_1_ vs. N_2_–N_3_	1.773	0.867–3.626	0.117			
Clinical stage						
I–II vs. III–IV	1.055	0.311–3.582	0.031	1.538	1.306–3.867	0.027
ZIP4 expression						
Low vs. high	2.185	0.958–4.236	0.01	2.315	1.421–5.229	0.012

### ZIP4 overexpression induces the EMT and ZIP4 downregulation represses the EMT phenotype of C666-1 cells in vitro

**Fig. 2:**
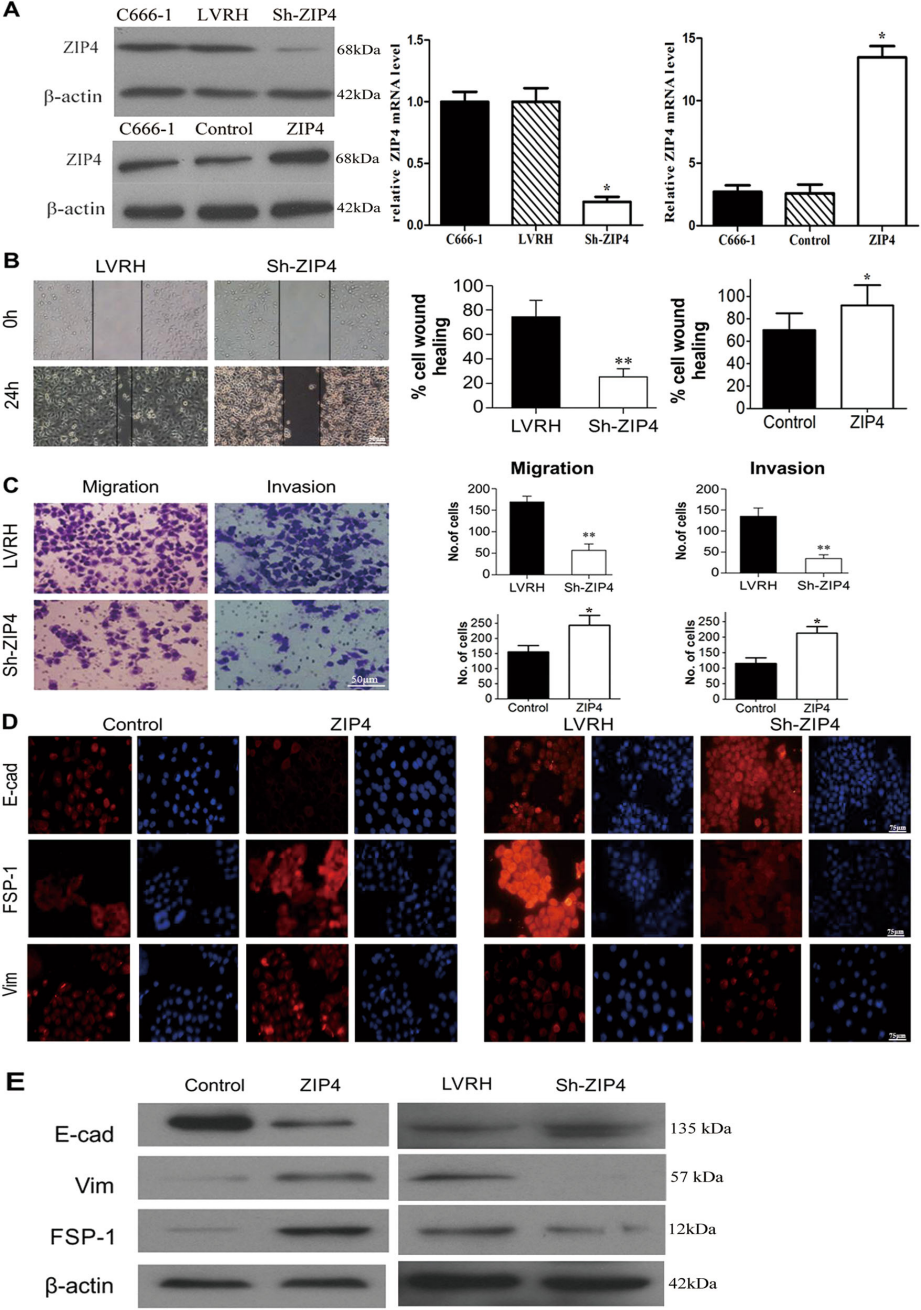
ZIP4 silencing inhibited migration and invasion capabilities of C666-1 cells. **a** Relative protein and mRNA level of ZIP4 were detected in stable LVRH, Sh-ZIP4, Control, and ZIP4 cell lines. **b** The cell migration rate between LVRH C666-1 and Sh-ZIP4 C666-1 cells was compared by the wound-healing assay. Microscopic observation was recorded at 0 and 24 h after scratching the cell layer (Scale bar = 50 μm). **c** The invasive and migration properties of the cells were analyzed by using a matrigel-coated Boyden chamber. Migrated cells were plotted as the average number of cells per field from three different experiments (Scale bar = 50 μm). **d** The expression levels of EMT markers, E-cadherin (E-cad), vimentin (Vim), and FSP-1 were examined by immunofluorescence staining (Scale bar = 75 μm). **e** E-cad, vimentin (Vim), and FSP-1 expression were examined in control and ZIP4 C666-1 cells by western blot. The experiments were performed in triplicate. The data are represented as means ± SD from three independent experiments. *P < 0.05

To further investigate the biological functions of ZIP4 in NPC, we first constructed lentivirus vectors to express a ZIP4 shRNA (Sh-ZIP4) and full-length human ZIP4 cDNA (ZIP4) in C666-1 cells. After 48 h of viral infection, more than 90% of the cells were RFP-positive, indicating a high efficiency of transfection. Stable cell lines expressing Sh-ZIP4, ZIP4, or empty vectors (LVRH and control) were selected by adding 0.5 μg/mL of puromycin to the medium. ZIP4 protein levels were determined by western blot and PCR in Sh-ZIP4 cells and ZIP4 C666-1 cells, which were compared with cells expressing the empty lentiviral vector (*P* < 0.05) (Fig. 2a).

Both in vitro scratch assays and matrigel transwell assays were utilized to study the effects of ZIP4 on migration and invasion of C666-1 cells. ZIP4 downregulation significantly inhibited migration and invasion of C666-1 cells. As shown in Fig. 2b, when endogenous ZIP4 was downregulated in C666-1 cells using specific shRNAs, the migration index measured by wound-healing assays decreased compared with that of vector control cells (LVRH). The same effect was observed in transwell assays as shown in Fig. 2c. Compared with the scrambled vector control, cell migration ability was decreased by ~70% in Sh-ZIP4 C666-1 cells, whereas their invasive ability was decreased by ~78%.

The EMT is a crucial step in the initiation of metastasis in tumor cells^24^. Thus, the effect of ZIP4 on the EMT phenotypes was determined in NPC cells. The immunofluorescence assay showed that ZIP4 overexpression in C666-1 cells led to downregulation of epithelial markers (E-cadherin) and upregulation of mesenchymal markers (FSP-1) and vimentin (Vim). However, in ZIP4 downregulated C666-1 cells, increased E-cadherin expression and reduced expression of FSP-1 and Vim were detected, indicating that ZIP4 knockdown induced the mesenchymal–epithelial transition in C666-1 cells (Fig. 2d). Likewise, western blotting analysis confirmed the expression of these markers in ZIP4-transfected and Sh-ZIP4-transfected C666-1 cells (Fig. 2e). Taken together, these data indicate that ZIP4 upregulation contributes to NPC tumorigenesis, as well as the migration and invasion of NPC cells.

### ZIP4 silencing inhibits C666-1 cell metastasis, dissemination, and invasion in vivo

**Fig. 3:**
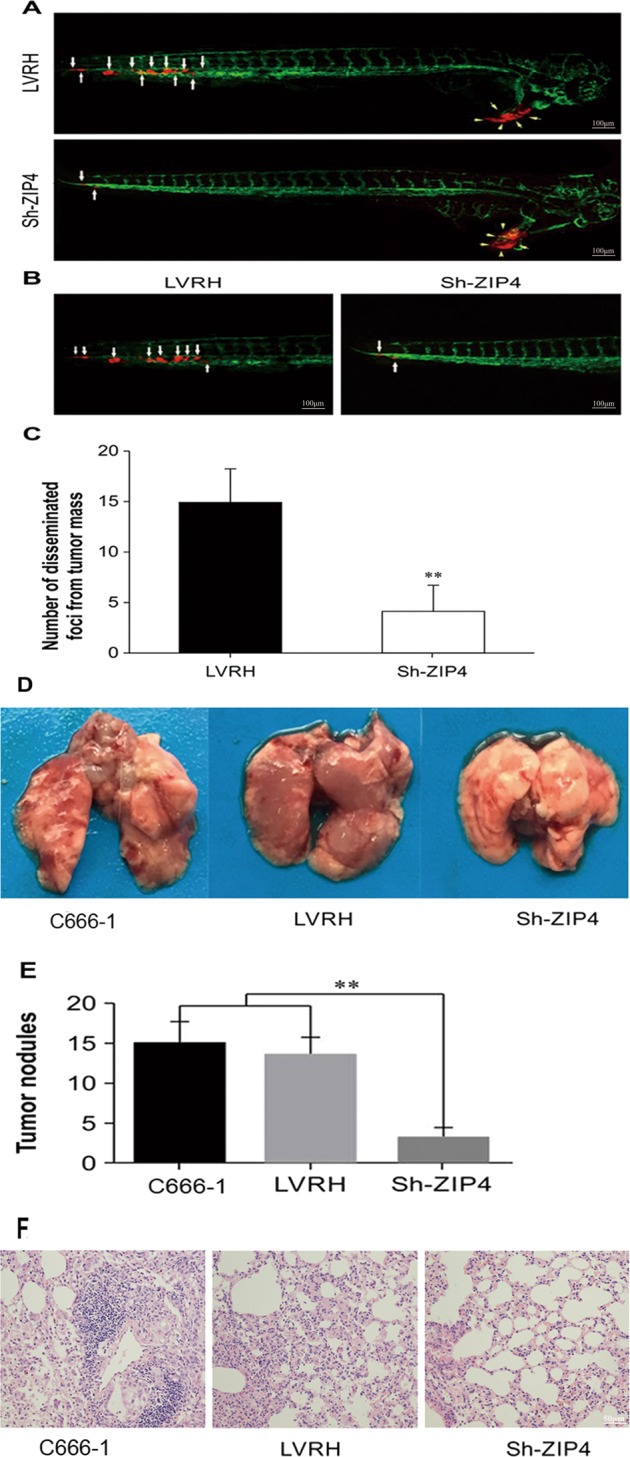
ZIP4 silencing prevents C666-1 tumor cell invasion, dissemination, and metastasis in a zebrafish model and a lung metastasis mouse model. **a**, **b** Red fluorescence-labeled tumor cells were injected into the perivitelline space of 48 h postfertilization embryos and tumor cell invasion, dissemination, and metastasis were detected using confocal microscopy at day 3 post injection. Yellow arrows indicate primary tumors. White arrows indicate disseminated tumor foci (Scale bar = 100 μm). **c** Quantification of disseminated tumor foci (*n* = 40 per group), *P* < 0.01. **d** Representative images of the mouse lungs. **e** The average number of tumor metastatic nodules in the lungs of mice xenografted with C666-1, LVRH C666-1 cells, and Sh*-*ZIP4 C666*-*1 cells is provided (*N* = 7; *P* < 0.01). **f** Representative views of lung tissue sections from each group are shown (Scale bar = 50 μm). The experiments were performed in triplicate. The data are represented as means ± SD from three independent experiments

Xenograft engraftment of tumor cells into optically clear zebrafish has allowed dynamic visualization and new mechanistic insights into the processes by which cancer cells migrate and disseminate throughout the body. So we labeled C666-1 cells with a red fluorescent protein, and then the cells were transplanted to flk1-EGFP fish that expressed green fluorescent protein in the vascular endothelial cells. The specific methods are as follows. Fifty to one hundred red fluorescence-labeled NPC cells (Sh-ZIP4 C666-1 cells or LVRH C666-1 cells) (Supplementary Fig. 1) were implanted in the perivitelline space of (hpf) Tg (flk1:EGFP) transgenic zebrafish embryos at 48 h post fertilization. Three days after cell injection, hemispheric solid tumors protruded from the abdomens of the Tg (flk1:EGFP) transgenic zebrafish. LVRH C666-1 cells were evidently disseminated away from the primary lesions, while most of the Sh-ZIP4 C666-1 cells remained at the primary sites. Only a few of the Sh-ZIP4 C666-1 cells were disseminated away from the primary sites (Fig. 3a). In tumor-bearing fish embryos, the size of the primary tumors was similar in both groups. In terms of local invasion, many LVRH C666-1 cells disseminated to distal parts of the fish body, including the tail (Fig. 3b). Quantification analysis showed that a significantly higher number of disseminated tumor foci were detected in the LVRH C666-1-injected zebrafish in comparison with the Sh-ZIP4 group (Fig. 3c). These results suggest that C666-1 cells metastasis, dissemination, and invasion were inhibited by ZIP4 silencing in transgenic zebrafish xenograft.

Furthermore, an in vivo metastasis assay was conducted in nude mice to demonstrate the effect of ZIP4 on tumor metastasis. Nude mice were euthanized on day 30 and their lungs were harvested. The number of pulmonary metastatic nodules induced by Sh-ZIP4 C666-1 cells was significantly lower than that of induced by LVRH C666-1 cells (Fig. 3d, e, *P* < 0.01). Both the size and number of lung micrometastatic nodes were remarkably decreased in mice injected with Sh-ZIP4 C666-1 cells in comparison with LVRH mice (Fig. 3f, *P* < 0.01), as demonstrated by hematoxylin and eosin (H&E) staining.

### ZIP4 affects the PI3K-Akt signaling pathway

**Fig. 4:**
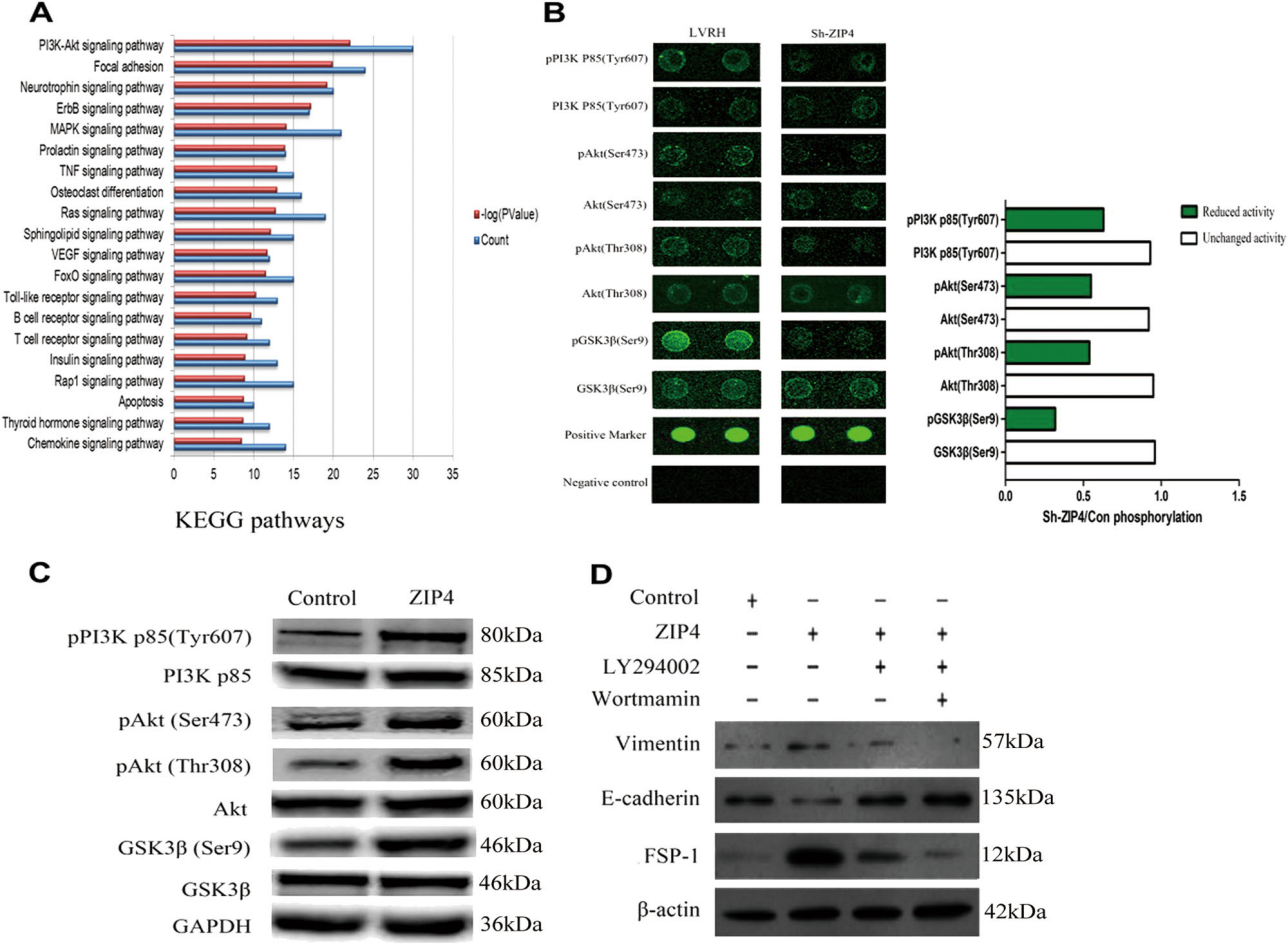
ZIP4 associated signaling pathways and mechanism were enriched. **a** KEGG pathway analysis of the discrepantly expressed genes in Sh-ZIP4 compared with control LVRH C666-1 cells. **b** Phospho-antibody array. See Supplementary Fig. 1 for full results and additional annotations. **c** The western blot assay of the selected proteins for the determination of phosphorylation antibody array. **d** Effect of PI3K inhibitors, LY294002, and Wortmannin on EMT markers. The experiments were performed in triplicate. The data are represented as means ± SD from three independent experiments. *P < 0.05

In stable ZIP4 knockdown C666-1 cells, a phospho-specific antibody microarray (PCS248) analysis was conducted to clarify the molecular mechanism by which ZIP4 promotes NPC progression and metastasis. A KEGG pathway analysis of the phospho-antibody array indicated that knockdown of ZIP4 mainly resulted in suppression of the PI3K-Akt (phosphatidylinositol-3-kinase/Akt) signaling pathway (Fig. 4a). Notably, in LVRH cells, we observed that knockdown of ZIP4 reduced the transcript levels of pPI3K p85 (Tyr607), pAkt (Ser473), pAkt (Thr308), and pGSK3β (Ser9) without altering their corresponding total protein levels (Fig. 4b and Supplementary Fig. 2). We further validated changes in the levels of these proteins in NPC cells with ZIP4 overexpression using western blotting. Targeted overexpression of ZIP4 led to increased phosphorylation of these four proteins in comparison with control NPC cells, while total protein levels remained unchanged (Fig. 4c). Moreover, to further investigate the mechanism underlying ZIP4-induced EMT, we determined that, after the addition of PI3K-Akt pathway inhibitors LY294002 and wortmannin, expression of EMT marker E-cadherin (epithelial cell markers) was upregulated, while expression of FSP-1 and Vim (mesenchymal cell marker) was downregulated (Fig. 4d). These results indicate that the PI3K-Akt signaling pathway is involved in promotion of the EMT via ZIP4 in NPC.

### ZIP4 silencing mediates C666-1 cell sensitivity to radiotherapy in vitro

**Fig. 5:**
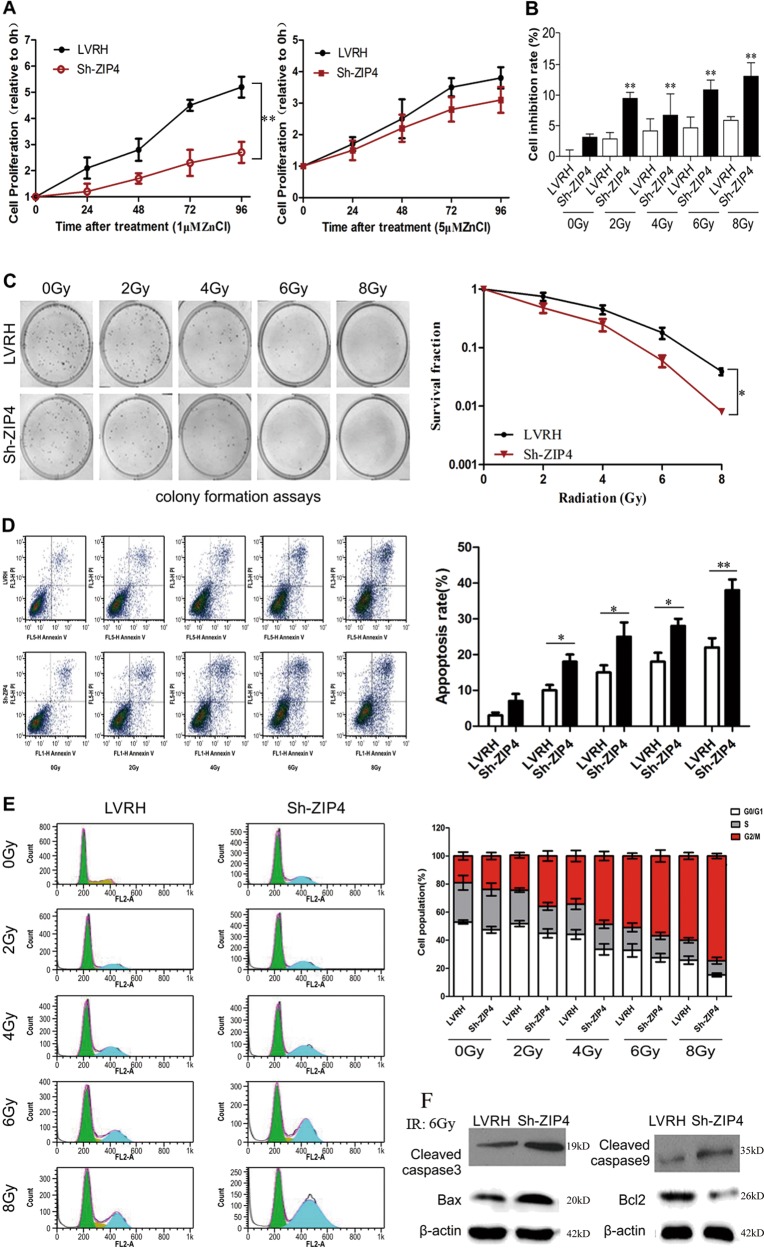
ZIP4 silencing enhances radiosensitization in vitro. **a** Cell growth in low and high concentration of zinc. **b** Inhibition of cell proliferation following exposure to various doses of Co^60^ was evaluated by the MTT assay (*P* < 0.01). **c** Images and quantification of the number of colonies formed from cells treated with 0, 2, 4, 6, and 8 Gy (*P* < 0.05). **d** Flow cytometry analysis of apoptosis using annexin V and propidium iodide in cells treated with various doses of radiation (0, 2, 4, 6, and 8 Gy). **e** Flow cytometric analysis of the cell cycle in cells treated with various doses of radiation. **f** Protein expression levels of cleaved caspase-3, cleaved caspase-9, Bax, and Bcl-2 in cells treated with a 6 Gy dose of radiation. Experiments were repeated three times. The data are represented as means ± SD from three independent experiments. *P < 0.05

To investigate whether silencing of ZIP4 affects the proliferation of NPC cells, cells were treated with TPEN to remove intercellular zinc, followed by culturing in medium with the indicated concentrations of ZnCl_2_ (0–10 μM). The growth of the LVRH cells was significantly greater than that of the Sh-ZIP4 cells after ZnCl_2_ treatment at a low zinc concentration (1 μM). There was no remarkable difference in the growth of LVRH and Sh-ZIP4 cells at the high zinc concentration (5 μM) because the high zinc concentration was toxic to the cells (Fig. 5a). Our results suggest that Sh-ZIP4 depressed the EMT phenotype of C666-1 cells. Previous studies demonstrated that the EMT induces resistance to radiotherapy or chemotherapy. Thus, we speculate that Sh-ZIP4 could affect radiosensitivity. To verify this hypothesis, we further explored proliferation and colony-formation abilities after irradiation (0, 2, 4, 6, and 8 Gy) in C666-1 cells. Thiazolyl blue tetrazolium bromide (MTT) assays indicated a significant decrease in the number of surviving Sh-ZIP4 C666-1 cells in comparison with LVRH control cells 48 h after irradiation (Fig. 5b). The number of cell colonies at all tested doses of radiation was significantly decreased in Sh-ZIP4 C666-1 cells in comparison with that of LVRH C666-1 cells (Fig. 5c).

In addition, cell death was assessed by flow cytometry. The results were similar, showing that more apoptotic cells were observed at every radiation dose in Sh-ZIP4 cells in comparison with that of LVRH C666-1 cells (Fig. 5d). As shown in Fig. 5e, radiation significantly disrupted the cell cycle progression and induced a dramatic increase in G2/M phase cells in comparison with nonirradiated C666-1 cells. No significant differences in the cell cycle profiles were observed between the ZIP4 knockdown and LVRH cells in the defect of radiation. On the contrary, at every radiation dose, significantly more Sh-ZIP4 C666-1 cells were arrested in the G2/M phase in comparison with LVRH C666-1 cells, and sh-ZIP4 C666-1 cells in the G1 phase were decreased. These data suggest that knockdown of ZIP4 enhanced radiation-induced arrest of the cell cycle in the G2/M phase. These results suggest that Sh-ZIP4 cells were more sensitive to radiotherapy than were LVRH C666-1 cells. Radiation-induced apoptosis in C666-1 cells was confirmed with western blotting. As shown in Fig. 5f, the expression of cleaved caspase-3, cleaved caspase-9, and Bax significantly increased, while Bcl-2 expression decreased in Sh-ZIP4 C666-1 cells compared with LVRH C666-1 cells exposed to 6 Gy radiation. Taken together, these results demonstrate that ZIP4 is a crucial regulator of therapeutic sensitivity in NPC cells.

### ZIP4 silencing enhances the sensitivity of human NPC cells to radiotherapy in vivo

**Fig. 6:**
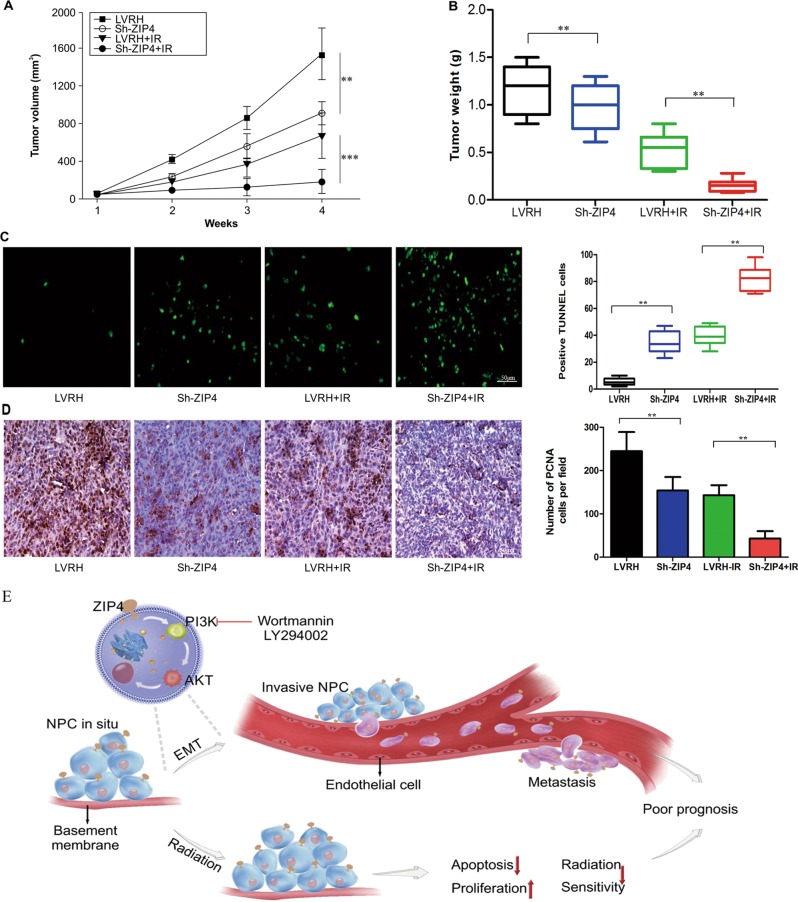
ZIP4 silencing enhances the antitumor effect of radiation in vivo and Schematic summary of this study. **a**, **b** Sh-ZIP4 and LVRH C666-1 cells (1 × 10^7^) were subcutaneously inoculated into the right hind limb of nude mice (*N* = 7 per group). When the size of the tumors reached about 50 mm^3^, tumors were irradiated at 5 Gy per day for 4 days (on day 1, 3, 6, and 8). Tumor size was measured and weight was recorded. **c** TUNEL staining of C666-1 xenografts (Scale bar = 50 μm) (*P* < 0.01). **d** PCNA IHC staining of C666-1 xenografts (*P* < 0.01). **e** Overexpression of ZIP4 induces the EMT process through the PI3K/Akt pathway and further promotes migration and invasion of NPC cells. Meanwhile, ZIP4 enhanced radiation-induced apoptosis and growth inhibition of human C666-1 cells. These findings support our clinical observations, indicating that ZIP4 elevation is associated with clinical T classification, N classification, and clinical stage, and leads to adverse survival outcomes of NPC

The combined antitumor effects of Sh-ZIP4 and radiation were determined in nude mice with C666-1 tumors. According to the tumor growth curves, tumor growth was suppressed in the Sh-ZIP4 group in comparison with the LVRH group. Furthermore, in comparison with that of the LVRH + IR group, a superior antitumor effect was observed in the combined treatment Sh-ZIP4 + IR group (Fig. 6a). Moreover, the Sh-ZIP4 + IR groups showed markedly reduced tumor weight in comparison with that of the LVRH + IR group (Fig. 6b), indicating the strong action of Sh-ZIP4 on the radiation sensitization of C666-1 cells.

To determine the mechanisms by which the antitumor effects of radiation were enhanced by ZIP4 knockdown in vivo, tumor sections from mice in each group were stained with terminal deoxynucleotidyl transferase dUTP nick-end labeling reagent and anti-PCNA antibodies to evaluate apoptosis and proliferation rates, respectively. More apoptotic cells were detected within the tumors treated with combining Sh-ZIP4 and radiation in comparison with the other treatments (Fig. 6c). Tumors from the LVRH group demonstrated high PCNA density, while a decrease in PCNA density was observed in those from the combined treatment group and the group treated with Sh-ZIP4 alone (Fig. 6d). These data suggest that Sh-ZIP4 can enhance apoptosis of tumor cells and inhibit tumor proliferation in vivo, consistent with the results observed in vitro, indicating that ZIP4 silencing has the potential to strongly enhance the antitumor effects of radiotherapy.

## Discussion

Zinc transporter ZIP4 is critical for tumor growth and progression, including invasiveness and metastasis, in various cancers. However, the role of ZIP4 in NPC pathogenesis and progression is largely unknown. Radiotherapy is the most reliable nonsurgical treatment for NPC. Radioresistance is the main obstacle limiting the benefits of radiotherapy because it can result in the recurrence and distant metastasis of tumors^25^. Therefore, we sought to identify the role of ZIP4 in regulating the invasive behavior and radiosensitivity of NPC.

Recent studies indicate that deregulated expression of ZIP4 is linked to pancreatic cancer, HCC, gliomas, and prostate carcinoma; however, the relationships between ZIP4 expression levels and clinical variables are unclear. First, a gene profile study revealed that ZIP4 is the only zinc transporter that is significantly upregulated in human pancreatic cancer^26^. Abnormal ZIP4 expression is associated with human pancreatic cancer nosogenesis and progression^15^, which indicates that ZIP4 is a novel diagnostic and prognostic marker for human pancreatic cancer^14,16,19^. Moreover, ZIP4 expression was significantly associated with tumor recurrence, tumor node metastasis stage, and tumor size in patients with HCC after liver transplantation. In addition, ZIP4 was an independent predictor of OS in these patients^21^. Another study found that elevated ZIP4 expression was correlated with shorter OS and higher glioma grade^22^. However, other studies found that ZIP4 expression was significantly downregulated in prostate carcinoma tissues and suggested that there was no correlation between ZIP4 expression and the pathologic grade of prostate carcinoma^23^. Very few studies have connected ZIP4 expression with NPC progression.

As far as we know, this is the first study to assess outcomes relative to ZIP4 expression levels in NPC patients. Our results indicate that elevated ZIP4 levels occurred more frequently in stage III + IV patients than in stage I + II patients. In addition, high ZIP4 expression was significantly associated with advanced tumor status and advanced TN stage. Moreover, in survival analysis, high ZIP4 expression predicted worse OS, RFS, and DMFS. More importantly, ZIP4 overexpression (HR = 2.315, *P* = 0.012) and clinical stage (HR = 1.538, *P* = 0.027) were independent predictors for OS in the multivariate analysis. These results indicate that ZIP4 may be a promising marker in NPC nosogenesis and prognosis

It is widely accepted that the EMT is crucial to malignant tumor metastasis and invasion^27^. Mesenchymal markers, such as Vim and FSP-1, are acquired during the EMT, while epithelial cell adhesion molecules, such as E-cadherin, are lost^28–30^. Previous studies reported that ZIP4 promotes invasion and metastasis in HCC and pancreatic carcinoma^15,21^. In contrast, ZIP4 inhibits prostate carcinoma cell proliferation and invasion. Since ZIP4 expression is positively correlated with TN classification and clinical stage, we speculated that ZIP4 induces invasiveness and migration in NPC cells by inducing the EMT. As expected, immunofluorescence showed that ZIP4 overexpression in C666-1 cells induced a decrease in the abundance of epithelial markers (E-cadherin) and an increase in the abundance of mesenchymal markers (FSP-1 and Vim). However, E-cadherin expression was increased in C666-1 cells with ZIP4 downregulation, while the expression levels of Vim and FSP-1 were reduced, which supports the role of ZIP4 in facilitating the EMT in NPC. Likewise, western blotting analysis confirmed these trends. To further investigate the signaling pathways underlying ZIP4-induced EMT, phospho-antibody arrays and western blot validation were carried out. According to the antibody arrays, PI3K-Akt signaling is involved in the functions of ZIP4. The addition of the PI3K-Akt pathway inhibitors, LY294002 and wortmannin, upregulated E-cadherin expression and down-regulated FSP-1 and Vim expression. These data indicate that the PI3K-Akt pathway is involved in the promotion of EMT by ZIP4. It is reasonable to speculate that some compound or reagent targeting ZIP4 and consequently inhibiting the PI3K/Akt signaling pathway may act as a novel therapeutic strategy for NPC.

We also constructed zebrafish xenograft model^31–35^ and mouse model to elucidate the effects of ZIP4 on invasion and metastasis in NPC. Owing to the zebrafish are transparent, which allows visually monitor metastasis behavior of tumor cells in real time^36^. Only a few cancer cells are needed to construct zebrafish xenografts and the rapid progress of metastasis in zebrafish xenografts can be observed within 48 h after microinjection, as well as large sample of zebrafish improve the effectiveness of statistical analyses^37^. Our current zebrafish model in combination with lung metastasis mouse model would be complementary and reveal that ZIP4 silencing in C666-1 cells prevents invasion, dissemination, and metastasis. Our results suggest that Sh-ZIP4 depressed the EMT phenotype of C666-1 cells, and previous studies demonstrate that the EMT induces resistance to radiotherapy or chemotherapy^38,39^. Thus, we speculate that ZIP4 downregulation should enhance the sensitivity of NPC to radiotherapy. We demonstrate that knockdown of ZIP4 in NPC C666-1 cells significantly promoted radiation-induced apoptosis and restrained tumor growth. Interestingly, we demonstrated that ZIP4 silencing significantly strengthens the antitumor effect of radiotherapy, contributing to tumor growth inhibition in our xenograft nude mouse model. These findings suggest that ZIP4 may be a novel potential therapeutic target that could be exploited to increase the radiosensitivity of human NPC.

In conclusion, our results demonstrate for the first time that ZIP4 is substantially overexpressed in advanced stage clinical NPC specimens and high expression levels of ZIP4 positively correlates with poor prognosis in patients with NPC. Moreover, we demonstrate that ZIP4 expression significantly regulates cell migration and invasion in vitro and in vivo, partially through the PI3K-Akt pathway. ZIP4 upregulation suppressed radiosensitivity, accelerated cell proliferation, and inhibited cell apoptosis (Fig. 6d). These data indicate novel functions for ZIP4. Though many aspects require further study, we speculate that ZIP4 may serve as a potentially useful diagnostic and prognostic marker for NPC, and combining ZIP4 depletion and radiation may represent a rational strategy for the treatment of patients with NPC.

## Materials and methods

### Reagents and antibodies

The MTT cell proliferation assay, propidium iodide, ribonuclease (RNase A), protease inhibitor cocktail, radioimmunoprecipitation assay buffer, bovine serum albumin, and Dulbecco’s phosphate buffered saline (PBS) were purchased from Sigma-Aldrich Co. (St Louis, MO, USA). Human ZIP4 antibodies were purchased from Proteintech (Rosemont, IL). PCNA and phospho-PI3 kinase p85 (Tyr607) antibodies were purchased from Abcam (Cambridge, MA, USA). Cleaved caspase-3, cleaved caspase-9, Bax, Bcl-2, E-cadherin, FSP-1, Vim, PI3 kinase p85, Akt, phospho-Akt (Ser473), phospho-Akt (Thr308), GSK3β, and phospho-GSK-3β (Ser9) antibodies were purchased from Cell Signaling Technology (Danvers, MA, USA). Human β-actin antibodies were purchased from Santa Cruz Biotechnology (Dallas, TX, USA). All antibodies were used according to the manufacturers’ instructions. PI3K inhibitors (LY294002 and wortmannin) were purchased from Sigma-Aldrich Co. The other chemicals used in this study were of analytical reagent grade.

### Cell lines, animals, immunohistochemistry, and western blot analysis

The human NPC C666-1 cell line was obtained from Shanghai Gaining Biological Technology Co., Ltd. The C666-1 cell line was authenticated by ShanghaiBiowing Applied Biotechnology Co., Ltd using short tandem repeat (STR) profiling. The STR profiles meet the standards recommended for C666-1 cell line authentication. The C666-1 cells tested negative for mycoplasma. 293T cells were purchased from the American Type Culture Collection (ATCC, Manassas, VA, USA), and authenticated by ATCC using STR profiling. Details concerning cell culture, animals, IHC, and western blot analysis can be found in the online supplementary methods.

IHC staining was evaluated using a semiquantitative scoring method. We graded each sample on the basis of the H-score (H-score = *I* × *P*, where *I* is the staining intensity score and *P* is the score for the percentage of cells at each staining intensity level). *I* was scored as follows: 0, no staining; 1, weak staining; 2, moderate staining; 3, strong staining. The positively stained area (*P*) was scored as follows: 0, <5%; 1, 5–25%; 2, 26–50%; 3, 51–75%; 4, >75%. ZIP4 protein positive staining score value from 0 to 12 was calculated for each NPC sample. The staining score of less than 6 indicated low ZIP4 expression, whereas the score greater than 6 indicated high ZIP4 expression. The relationships between positive ZIP4 immunostaining in lesion tissues and clinicopathological parameters were determined.

### Patients and treatment

This research was approved by the Ethics Committee of Sichuan Cancer Hospital (Chengdu, China). Ninety-nine patients with unabridged medical records were recruited for the study. All patients were restaged based on the 8th edition of the American joint committee on cancer (AJCC) staging system^40^. The clinical characteristics and ZIP4 expression of the NPC patients are described in Table 1. All patients were treated with standard curative radiotherapy with or without chemotherapy^41^. All patients also underwent concurrent cisplatin-based chemotherapy. Written informed consent of the study was obtained from all NPC patients. We covered patient treatment and follow-up in more detail in the supplementary methods.

### Stable cell line selection

Stable cells expressing ZIP4 shRNA were selected from C666-1 cells infected with lentivirus vectors following the manufacturer’s instructions. ZIP4 shRNA (Sh-RNA; 5′-ACGTAGCACTCTGCGACATGGTCAGGATG-3′) was used to downregulate ZIP4 expression. Synthesis of lentiviral supernatants was conducted by GeneCopoeia (Guangzhou, China). C666-1 cells were infected with viral supernatants. Puromycin (0.5 μg/mL) was added to the medium for the selection of stable cell lines expressing Sh-ZIP4 or psi-LVRH1MP (LVRH). Stable cells overexpressing ZIP4 were selected from C666-1 cells infected with a lentivirus vector following the manufacturer’s directions. The full-length human ZIP4 cDNA was inserted into lentiviral vector CD510B-1 (control), and then lentiviral packaging plasmids (PMD2.G and psPAX2) and the recombinant plasmid were cotransfected into 293T cells. Viral supernatants were collected and used to infect C666-1 cells. Stable cell lines expressing ZIP4 and control cells were selected by adding 0.5 μg/mL of puromycin in the medium.

### Cell viability, migration, invasion assays, apoptosis assay, and cell cycle analysis

Cell viability, migration, invasion, apoptosis assay, and cell cycle analysis for NPC cells in vitro were performed as described in the supplementary methods.

### Effects of zinc on cell growth

Growth assays were conducted in LVRH and Sh-ZIP4 C666-1 cells after treatment with ZnCl_2_ to determine the impact of zinc on cell proliferation. Each group cells (2 × 10^3^ cells/well) were starved in serum-free media for 24 h. The cells were given with 4 μM TPEN (*N,N,N*′*,N*′-tetrakis (2-pyridylmethyl)ethylenediamine) to co-cultured 1 h at 37 °C. After washing with PBS, the cells were incubated in DMEM containing 0–5 mM ZnCl_2_ for the indicated periods of time. Cell growth was assessed 24, 48, 72, and 96 h after serum starvation. The optical density at 490 nm was recorded using a microplate reader.

### Immunofluorescence staining

Cells were fixed with 4% paraformaldehyde and then washed with PBS for three times. Normal goat serum was incubated for 40 min, remove the blocking solution, and then add the primary antibody (anti-E-cadherin, anti-Vim, and anti-FSP-1 antibodies) overnight at 4 °C. Following incubation with the primary antibody, cells were washed three times with PBS and stained with the Cy3-conjugated secondary antibody for 30 min at 37 °C. The nuclei were stained with Hoechst 33258. Microscopy was performed after two washes with PBS. All immunofluorescence images were captured on an Olympus BX51 microscope and a DP 50 camera (Olympus). Images were handled by DPC controller software (Olympus).

### Zebrafish cell microinjection and imaging

In the past decade, zebrafish have emerged as a useful vertebrate model system to study the metastatic cascade in vivo. Zebrafish embryos are transparent and can be used to watch tumor invasion and spread in real time when human tumors were transplanted into them. So C666 cells have been employed to evaluate in vivo migration in zebrafish xenografts. Tg (flk1: EGFP) transgenic zebrafish carrying green fluorescent were used in all of our experiments. Each experiment was repeated at least three times, and 50 embryos were used in each experimental group. Red fluorescence-labeled cells (Sh-ZIP4 C666-1 and LVRH C666-1) were harvested at a concentration of 1 × 10^7^ cells/mL. Dechorionated embryos were collected 48 h after fertilization and anesthetized with 0.04 mg/mL tricaine (Sigma-Aldrich Co.). Suspended cells (5–10 nL containing ~50–100 cells) were implanted into the perivitelline space of each zebrafish embryo using an electronically regulated air–pressure microinjector (PL1-90; Harvard Apparatus, Holliston, MA, USA). Washed Zebrafish embryos with fish water and examined for the appearance of red fluorescent cells. Approximately 40 fish per implantation were transferred into six-well plates containing 2 mL of fresh fish water. Tg (flk1: EGFP) zebrafish embryos were maintained under normal fish husbandry conditions for 48 h with daily water changes. Invasion, dissemination, and metastasis of Sh-ZIP4 C666-1 and LVRH C666-1 cells were monitored daily in live zebrafish embryos.

Three days after implantation, zebrafish embryos were anesthetized (0.003% tricaine) and embedded into 3% methylcellulose in ventral, dorsal, and lateral orientations. A fluorescence microscope (Zeiss Imager.Z1, Carl Zeiss Microimaging Inc., Jena, Germany) equipped with an AxioCam MRc5 digital CCD camera (Carl Zeiss Microimaging Inc.) was used to acquire digital micrographs. A Zeiss Stemi 2000-C stereomicroscope with an AxioCam MRc5 digital CCD camera (Carl Zeiss Microimaging Inc.) was used to obtain whole animal images. Images were acquired in the same focal plane with either a bright field or light transmitted through RFP or GFP filters. ZEISS Axiovision rel.4.8 software was utilized for image capture, processing, and adjustment.

### Establishment of C666-1 lung metastasis model

Xenograft experiments were approved by the Laboratory Animal Ethical Committee at Sichuan University. To investigate the effect of ZIP4 on migration and invasion in vivo, a C666-1 lung metastasis model was established. Female nude mice (6–8 weeks old) were obtained from the experimental animal center of Sichuan University (Chengdu, Sichuan Province, China). To create the C666-1 lung metastasis mouse model, the nude mice were intravenously inoculated with 1 × 10^6^ C666-1 cells (C666-1, LVRH C666-1, and Sh-ZIP4 C666-1). Following euthanasia on day 30, lungs were harvested and weighed, and the pulmonary metastatic nodules were counted. Lungs were fixed in Bouin’s solution. Paraffin-embedded pulmonary sections were H&E stained. Histopathology examinations were accomplished using an Olympus microscope.

### Establishment of the C666-1 xenograft nude mice model

To create the C666-1 xenograft mouse model, female nude mice (6–8 weeks old) were subcutaneously injected in the right hind limb with 1 × 10^7^ C666-1 cells (Sh-ZIP4 C666-1 and LVRH C666-1 cells). While the tumors were ~50 mm^3^, the mice were treated with radiation or without radiation, respectively. The radiation treatment consisted of 5 Gy per day on days 1, 3, 6, and 8. Tumor size was measured every 2 days using calipers. Measurements were taken across the largest and perpendicular diameters. Tumor volume was calculated using the following formula: V = 0.52*AB*^2^, where *A* is the largest superficial diameter and *B* is the smallest superficial diameter.

### Phospho-specific protein microarray analysis

The microarray analysis was performed by Wanyen Biotechnologies Inc. (Shanghai). LVRH and Sh-ZIP4 cell lysates were used in a Phospho Explorer Antibody Array (PCS248, Wanyen Biotechnologies Inc.). The antibody array was composed of 269 antibodies with six replicates each. A majority of the antibodies (131) were phosphoproteins and their unphosphorylated counterparts. Antibody array slides were read using GenePix 4000B (Axon Instruments, USA). The following formula was used to calculate the phosphorylation ratio: phosphorylation ratio = phosphoE/unphosphoE, where phosphoE is the signal of phosphorylated protein and unphosphoE is the signal of the corresponding unphosphorylated protein. Lastly, the experimental data were analyzed by GenePix Pro 6.0 (Axon Instruments, USA). Phosphoproteins (*p* < 0.05) that were upregulated or downregulated by more than 1.3-fold were included in the analysis. The key signaling pathways were analyzed further using the DAVID database.

### Statistical analysis

Statistical analyses were performed with SPSS (v15.0, SPSS, Chicago, IL. USA) and GraphPad Prism 5.0 software (GraphPad, San Diego, CA, USA). All experiments were repeated three times in vitro. The results are shown as mean ± SD. Statistical differences between groups were determined with one-way analysis of variance. Chi-square and Fisher’s exact tests were performed to statistically analyze the relationships between clinicopathological parameters and ZIP4 expression in NPC. DMFS, RFS, and OS were defined as the time from the date of radiotherapy until the first date of documented distant metastasis, disease recurrence, or death from any cause, respectively. The Kaplan–Meier statistics and log-rank testing were utilized to evaluate the impact of the different clinical factors associated with OS, DMFS, and RFS rates. *P*-values < 0.05 were defined as statistically significant.

## Supplementary information

Supplementary Figure 1 and 2

Supplementary methods

C666-1 Cell Line Authentication

C666-1 the certificate

293T Cell Line Authentication

293T the certificate
