# Supplementary material for: Inhibition of ZIP4 reverses epithelial-to-mesenchymal transition and enhances the radiosensitivity in human nasopharyngeal carcinoma cells
Source: Cell Death Dis. 2019 Aug 5;10(8):588. doi: 10.1038/s41419-019-1807-7 (PMC6683154; doi:10.1038/s41419-019-1807-7)
Supplement: Supplementary file 1 — Supplementary Figure 1 and 2 [file 41419_2019_1807_MOESM1_ESM.doc]

**Supplementary Figure 1 and 2**

**
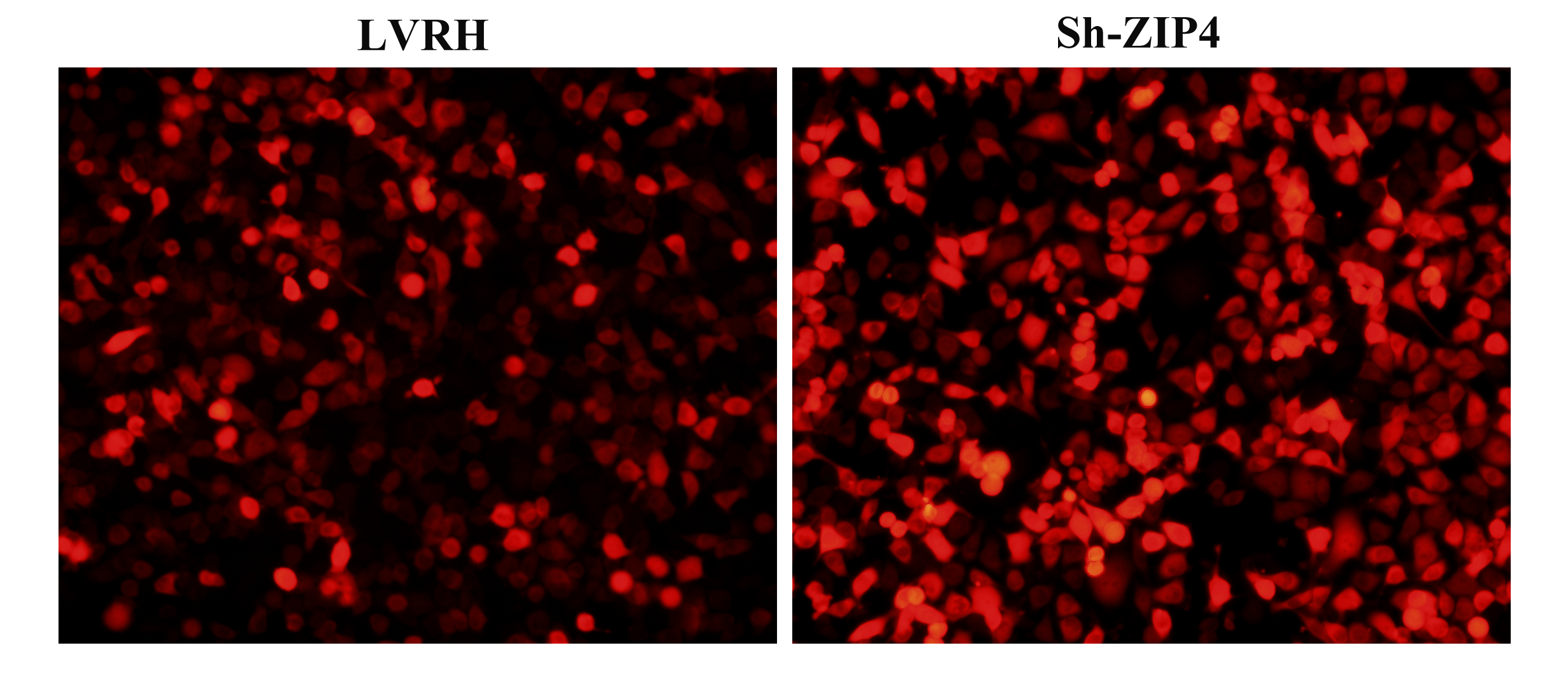
**

**Supplementary Figure 1:** Red fluorescence-labeled NPC cells (Sh-ZIP4 C666-1 cells or LVRH C666-1 cells). Viral supernatants were collected and used to infect C666-1 cells. Virus specific red fluorescence was observed between 48-72 hrs infection using a Leica inverted microscope.

**
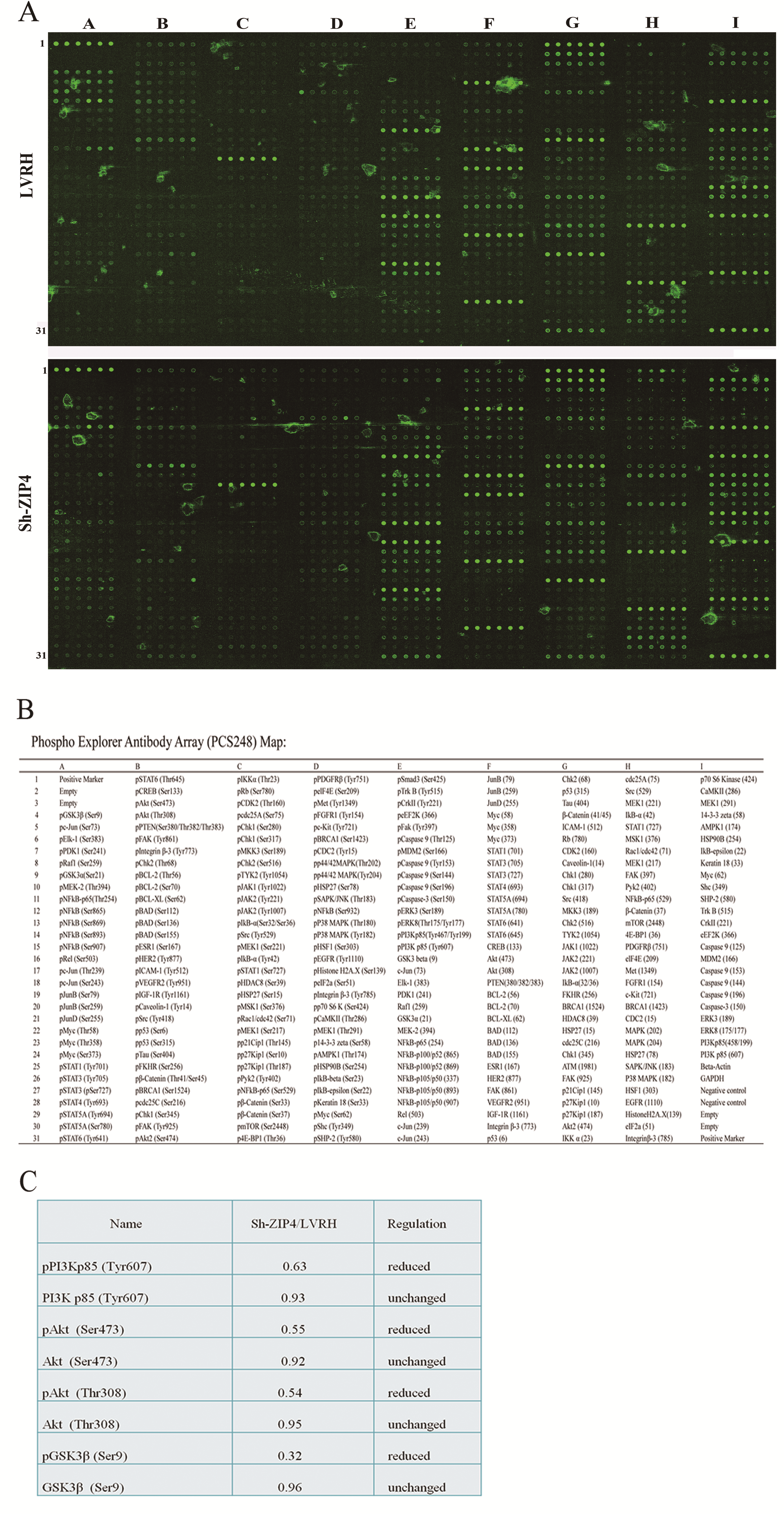
**

**Supplementary Figure2: Analysis of phospho-antibody array.** (A) Cell lysates obtained from LVRH and Sh-ZIP4 C666-1 cells, the phosphorylation states of proteins were then detected using antibody-based arrays. (B) Phospho Explorer Antibody array map. The locations of antibodies on the array are indicated. (C) The selected eight proteins are listed.
