# Supplementary material for: Inhibition of ZIP4 reverses epithelial-to-mesenchymal transition and enhances the radiosensitivity in human nasopharyngeal carcinoma cells
Source: Cell Death Dis. 2019 Aug 5;10(8):588. doi: 10.1038/s41419-019-1807-7 (PMC6683154; doi:10.1038/s41419-019-1807-7)
Supplement: Supplementary file 2 — Supplementary methods [file 41419_2019_1807_MOESM2_ESM.doc]

**Supplementary methods**

**Cell culture and animals**

All cell lines were incubated in a humidified chamber with 5% CO2 at 37°C. Tg(flk1: EGFP) zebrafish were bred and maintained normally (Temperature, 28°C; pH 7.2–7.4; 14 h on and 10 h off light cycle). Male 4–6-week-old nude mice (The laboratory Animal Center of Sichuan University, Chengdu, China) were kept under specific pathogen-free conditions in State Key Laboratory of Biotherapy and Cancer Center, West China Hospital, Sichuan University.

**IHC**

Immunohistochemical staining was performed on 4 µm thick sections that were sliced serially from paraffin-embedded blocks after formalin fixation of the pathological specimens. The sections were deparaffinized and rehydrated through a graded alcohol series (100, 95, 90, 85, and 75%). For antigen retrieval, the sections were immersed in 10 mM citric acid buffer (pH 6.0) autoclaved at 121°C for 5 min. After several washing steps in PBS, the sections were immersed in a solution of H_2_O_2_ (3%) to block endogenous peroxidase activity. Non-specific protein binding was attenuated by incubation for 30 min with goat serum. The sections were then incubated with a primary polyclonal rabbit anti-human ZIP4 antibody and a mouse anti-human PCNA antibody (Abcam; dilution, 1:100) overnight at 4°C. The sections were rinsed twice for 10 min with PBS and incubated with HRP-conjugated goat anti-rabbit IgG for 30 min, and then visualized using 3,3′-diaminobenzidine (DAB). Counterstaining was carried out with Mayer's hematoxylin. Tissue samples, to which no primary antibody had been added, were used as negative controls. The immunohistochemical staining was evaluated using a semiquantitative scoring method. Each sample was given a score according to the H-score. H-score = I × P. “I” means staining intensity. Score 0, no staining; score 1+, weak staining; score 2+, moderate staining; score 3+, strong staining. “P” means percent of cells at each staining intensity level. The positively stained area was scored as: <5% (0), 5–25% (1), 26–50% (2), 51–75% (3), and >75% (4). An individual ZIP4 IHC score (H-score) value from 0 to 12 was generated from the tumor sample of each patient. If the final immunoreactive score was less than 6, ZIP4 expression was considered as low. In contrast, if the score exceeded 6, ZIP4 expression was considered as high. Positive ZIP4 immunostaining in lesion tissues and its associations with clinicopathological parameters were analyzed.

**Western blot analysis**

For western blot analysis, cells were lysed on ice for 30 min with RIPA lysis buffer. Protein concentrations were determined by using the BCA assay. Fifty micrograms of total protein were loaded in each well and subjected to 12% sodium dodecyl sulfate-polyacrylamide gel electrophoresis and electronically transferred onto a polyvinylidene difluoride membrane. The membrane was incubated with 5% non-fat milk in TBST (10 mM Tris-HCl (pH 8.0), 150 mM NaCl, and 0.1% Tween-20) to block nonspecific binding sites for 2 h. The membranes were incubated with the primary antibodies against ZIP4, E-cadherin, vimentin, FSP-1, Bcl-2, Bax, cleaved caspase 3, and cleaved caspase 9 at the recommended dilution overnight at 4°C, washed three times with 0.1% Tween 20-TBS, and incubated with a HRP-linked secondary antibody (1:2000) for 1 h at room temperature. The membrane was washed three times with 0.1% Tween 20-TBS and the immunoreactive bands were detected by using a chemiluminescence detection kit (ECL; Amersham, Little Chalfont, UK).

**Cell viability assays**

LVRH C666-1 and Sh-ZIP4 C666-1 cells were seeded in 96-well culture plates (200 μL/well) at a density of 3000 cells/well in triplicate post-irradiation. After 12 h, the cells were treated with radiation at various doses (0, 2, 4, 6, and 8 Gy) and then incubated for another 72 h. Twenty microliters of MTT solution (5 mg/mL, Amresco, Solon, Ohio, USA) was added into each well and incubated for 4 h at 37°C. The culture medium was discarded, followed by addition of 150 μL of dimethyl sulfoxide (DMSO) and shaking for 10 min. The optical density (OD) at 570 nm was recorded by using a microplate reader.

For the colony formation assay, cells were plated at 500 cells per well in a 6-well plate (Corning, Corning, NY, USA) after being irradiated at various doses. Cells were allowed to proliferate in the culture medium for 10 days, with fresh medium replacement every 3 days. Colonies were washed with PBS, fixed with methanol, and stained with crystal violet.

**Cell migration and invasion assay**

The migration and invasion assays were performed using a Boyden chamber assay and would healing assay. Uncoated or coated with matrigel polycarbonate inserts of 8 μm pore size were used. Cells growing in the log phase were trypsinized, resuspended in serum-free medium, and seeded in the Boyden chambers. The top chambers of the transwells were filled with 200 μL of cells (4 × 10^5^ cells/mL) in serum-free medium and the bottom chambers were filled with 500 μL of RPMI 1640 medium containing 20% FBS. The cells were allowed to migrate for 24 h, at 37°C and 5% CO2. The cells from the upper surface of the filter were removed with a cotton swab and the cells that migrated through the filter were fixed with 4% paraformaldehyde. After 10 min, 0.1% crystal violet was used to stain the cells for 30 min. The number of cells that migrated through the inserts or invaded through the matrigel was determined in 5 randomly chosen visual fields.

For wound-healing assay, cells were seeded at a density of 3 × 10^5^ cells in 6-well plates for 12 h in RPMI 1640 culture medium with 10% FBS. When cells reached 90% confluence in 6-well plates, wounds were generated by scratching the monolayers with a 200 μl sterile plastic tip. Cells were washed to remove the detached cells and then maintained in RPMI 1640 medium without serum. The wound distances were measured at different time points (0, 3, 6, 9, 12, and 24 h) under the microscope. Wound healing percentage was calculated at the indicated time from acquired images.

**Apoptosis assay**

After treatment of the LVRH C666-1 and Sh-ZIP4 C666-1 cells with radiation at various doses (0, 2, 4, 6, and 8 Gy) and incubation for 48 h, the cells were harvested, washed twice with cold PBS, and resuspended in 100 μL of annexin V-FITC binding buffer. Cells were stained with 5 μL of annexin V-FITC and incubated in the dark at 4°C for 15 min followed by addition of 10 μL of PI. The samples were incubated for 15 min in the same conditions and then subjected to flow cytometry evaluation. This experiment was repeated three times. To assess the level of apoptosis in the C666-1 tumors, TUNEL assay was performed on tumor sections according to the manufacturer’s instructions (Promega, Madison, WI, USA).

**Cell cycle analysis**

The LVRH C666-1 and Sh-ZIP4 C666-1 cells were exposed to radiation at various doses (0, 2, 4, 6, and 8 Gy), cultured for 48 h, and then harvested. After rinsing twice with cold PBS and overnight fixation in 70% (v/v) ethanol at −20°C, the cells were centrifuged, washed once, resuspended in 200 μL cold PBS, treated with RNase for 30 min at 37°C in a water bath, and then filtered and stained with 400 μL of PI for 1 h at 4°C in the dark. Finally, the samples were analyzed using a BD FACS Calibur flow cytometer and the data were analyzed by using the FlowJo software.

**Patients and treatment**

This research was approved by the Ethics Committee of Sichuan Cancer Hospital (Chengdu, China). Ninety-nine patients with unabridged medical records were recruited for the study. The patient population composed of 77 males and 22 females with an age range of 22 to 81 years (mean age ± standard deviation, 46.7 ± 10.43 years). All patients were treated at Sichuan Cancer Hospital (Chengdu, China) between 2007 and 2010. All patients received a complete physical examination, endoscopy, CT and MRI of the head and neck, chest radiography, bone emission CT, and dental assessment before treatment. A nasopharyngeal neoplasm biopsy was performed to confirm the NPC diagnosis. The UICC 2002 staging system was utilized to stage the patients. The characteristics and distribution of the patients are listed in Table 1. All tumor tissue samples were fixed in 10% formalin, embedded in paraffin, and cut into 4-μm-thick sections. All patients were treated with standard curative radiotherapy with or without chemotherapy [34]. All patients also underwent concurrent cisplatin-based chemotherapy. Supplementary chemotherapy regimens were the same as those used for induction chemotherapy. Written informed consent was obtained from all patients.

**Patient follow-up**

All patients were followed for 3–6 years. Patients had one follow-up session every 3 months during year one, every 6 months during years two and three, and annually after year three. The follow-up sessions included indirect nasopharyngoscopy, nasopharyngeal and neck computed tomography/magnetic resonance imaging, abdominal B-mode ultrasonography, chest radiography, and blood tests.
