# Supplementary material for: Inhibition of ZIP4 reverses epithelial-to-mesenchymal transition and enhances the radiosensitivity in human nasopharyngeal carcinoma cells
Source: Cell Death Dis. 2019 Aug 5;10(8):588. doi: 10.1038/s41419-019-1807-7 (PMC6683154; doi:10.1038/s41419-019-1807-7)
Supplement: Supplementary file 3 — C666-1 Cell Line Authentication [file 41419_2019_1807_MOESM3_ESM.pdf]

# ShangHai BIOWING APPLIED BIOTECHNOLOGY Co. LTD

## Cell Line Authentication – STR Profiling

Sample Type: Cell Line

Sample from: Gaining Biological

Testing Method: STR Genotyping

Report Time:November 22, 2017

# COMPANY STATEMENT

1. THIS REPORT IS ONLY RESPONSIBLE FOR THE SAMPLES ANALYZED.
2. THE TESTING RESULTS AND THE ORGANIZATION NAME WILL NOT BE USED FOR ADVERTISEMENT, COMMERCIAL EXHIBITIONS, COMMERCIAL PERFORMANCE AND OTHER COMMERCIAL ACTIVITIES.
3. OBJECTIONS SHOULD BE RAISED WITHIN FIFTEEN DAYS AFTER THE RECEIPT OF THIS REPORT.
4. THE PAPER REPORT WITH CONTENT ALTERING, ADDING OR WITHOUT THE STAMPED SEAL OF THE COMPANY ARE INVALID.

**Testing Company:** Shanghai Biowing Applied Biotechnology Co. Ltd

**Address:** Room 4F, 8 th Buiding,Guiguo Garden,NO.471 Guiping Road,Caohejing Development Zone,Shanghai

**Tel:** +86-021-33559491

**Contact:** YiQun Chen

**E-mail:** biowing@vip.163.com

# Cell Line Authentication – STR Profiling Report

Sample code

Table 1. Sample Code

| Customer's code | Company Code |
|---|---|
| C666-1 | 20171114-05 |

Sample Number:1

Sample Type: Cell line

Testing Type: STR

Sample From: Gaining Biological

Testing Method:

DNA was extracted by a commercial kit from CORNING (AP-EMN-BL-GDNA-250G). The twenty STRs including Amelogenin locus were amplified by six multiplex PCR and separated on ABI 3730XL Genetic Analyzer. The signals were then analyzed by the software GeneMapper.

Test Results:

**1. Result**

Table 2. Matching information on the cell lines

| Sample Code | Multi-allele | Cell line matched | Cell Bank | Percentage |
|---|---|---|---|---|
| 20171114-05 | YES | C666-1 | EXPASY | 18/19 |

- **Multi-allele means some STR contain more than two loci.**

**2. Sample Description**

20171114-05: The DNA of the cell lines found to substantially match the type of cell lines in a cell line retrieval,EXPASY database shows that cells called **C666-1**，**CVCL_7949**corresponding to the cell number.

## 3. Genotyping Result

Table 3. STR and Amelogenin Genotyping Results of Cell line 20171114-05

| Marker | Sample | | | | Cell Bank information | | |
|---|---|---|---|---|---|---|---|
| | Allele1 | Allele2 | Allele3 | Allele4 | Allele1 | Allele2 | Allele3 |
| D5S818 | 11 | 11 | | | 11 | 12 | |
| D13S317 | 8 | 11 | | | 8 | 11 | |
| D7S820 | 11 | 12 | | | 11 | 12 | |
| D16S539 | 10 | 10 | | | 10 | 10 | |
| VWA | 17 | 18 | | | 17 | 18 | |
| TH01 | 6 | 8 | | | 6 | 8 | |
| AMEL | X | Y | | | X | Y | |
| TPOX | 8 | 11 | | | 8 | 11 | |
| CSF1PO | 11 | 15 | 16 | | 11 | 15 | 16 |
| D12S391 | 23 | 23 | | | | | |
| FGA | 23.2 | 23.2 | | | | | |
| D2S1338 | 16 | 22 | | | | | |
| D21S11 | 28 | 31.2 | | | | | |
| D18S51 | 15 | 16 | | | | | |
| D8S1179 | 11 | 16 | | | | | |
| D3S1358 | 16 | 17 | | | | | |
| D6S1043 | 19 | 19 | | | | | |
| PENTAE | 11 | 15 | | | | | |
| D19S433 | 12 | 15.2 | | | | | |
| PENTAD | 8.2 | 9.1 | | | | | |

Others:

1. Genotyping Strategy and Site Distribution

Attached Table. Experimental Strategy and Sites

| | Strategy 1 | Strategy 2 | Strategy 3 | Strategy 4 |
|---|---|---|---|---|
| 1 | TH01 | TPOX | D3S1358 | AMEL |
| 2 | D12S391 | VWA | D13S317 | D5S818 |
| 3 | D7S820 | D8S1179 | D6S1043 | D2S1338 |
| 4 | CSF1PO | PENTAD | D16S539 | D21S11 |
| 5 | FGA | | D19S433 | D18S51 |
| 6 | PENTAE | | | |

Technician: Jianan Zhang
Check: Lijin Gong
Person in Charge: Yiqun Chen

Issue date:November 22, 2017
