# Supplementary material for: Inhibition of ZIP4 reverses epithelial-to-mesenchymal transition and enhances the radiosensitivity in human nasopharyngeal carcinoma cells
Source: Cell Death Dis. 2019 Aug 5;10(8):588. doi: 10.1038/s41419-019-1807-7 (PMC6683154; doi:10.1038/s41419-019-1807-7)

Sample File	Sample Name	Panel	SQI	OS	SQ
05_E01_CellLineAuthentication1-10-1116.FSA	05	STR Profile 1-human			

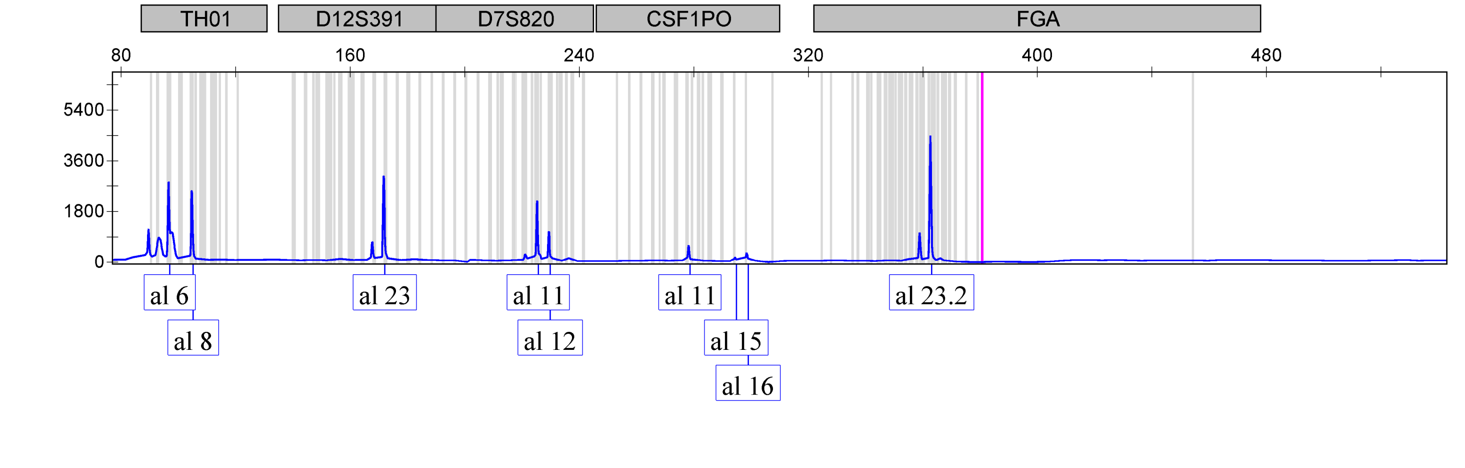

Sample File	Sample Name	Panel	SQI	OS	SQ
05_E01_CellLineAuthentication1-10-1116.FSA	05	STR Profile 1-human			

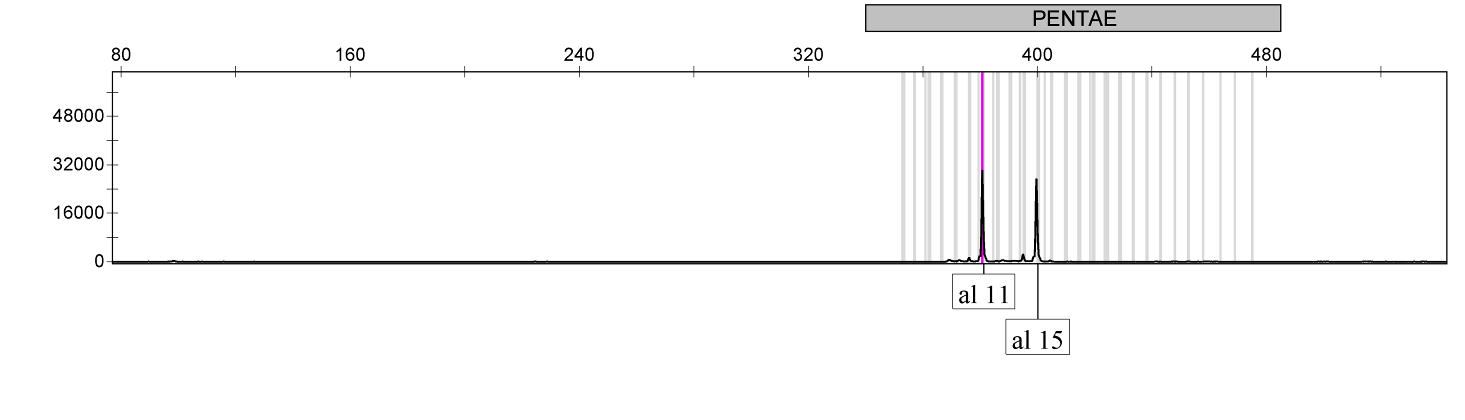

Sample File	Sample Name	Panel	SQI	OS	SQ
13_E02_CellLineAuthentication1-10-1116.FSA	05	STR Profile 2-human			

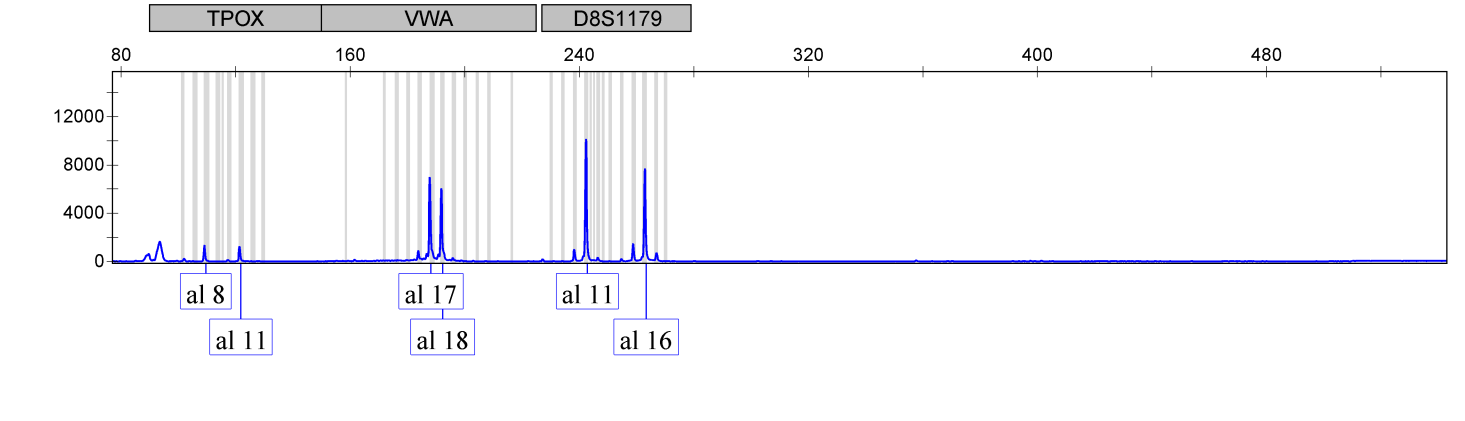

Sample File	Sample Name	Panel	SQI	OS	SQ
13_E02_CellLineAuthentication1-10-1116.FSA	05	STR Profile 2-human			

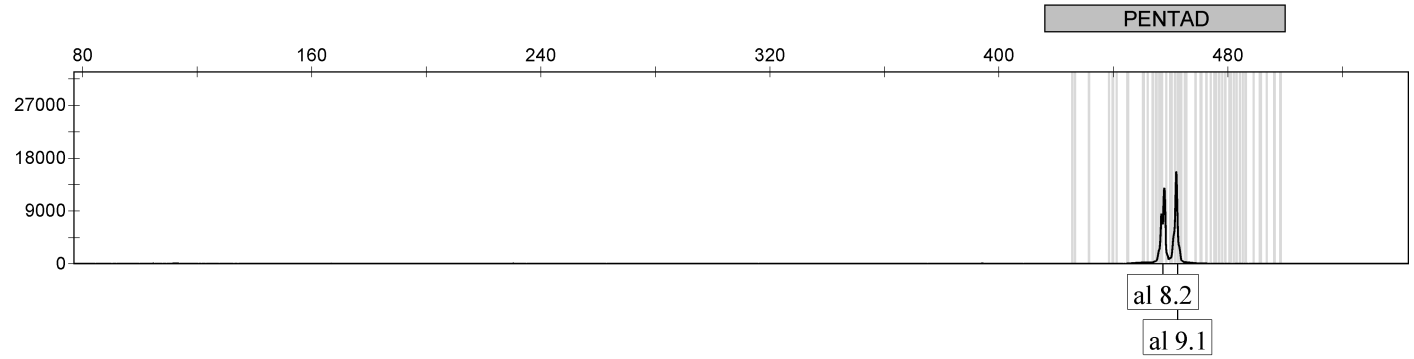

Sample File	Sample Name	Panel	SQI	OS	SQ
21_E03_CellLineAuthentication1-10-1116.FSA	05	STR Profile 3-human			

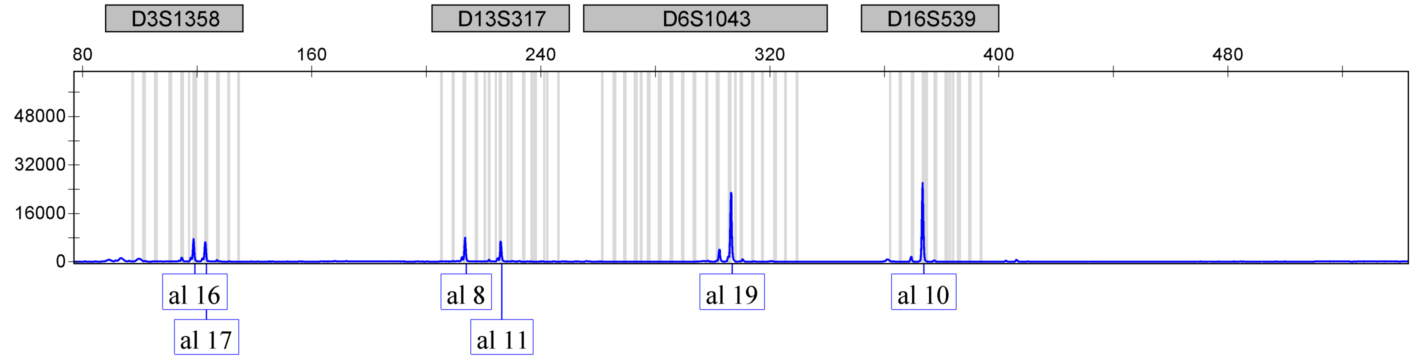

Sample File	Sample Name	Panel	SQI	OS	SQ
21_E03_CellLineAuthentication1-10-1116.FSA	05	STR Profile 3-human			

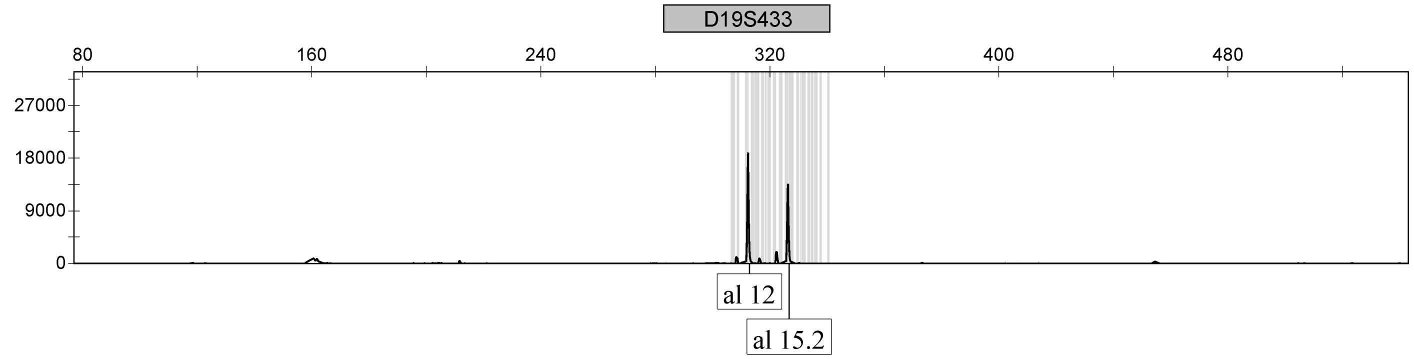

Applied Biosystems
GeneMapper 4.0

CellLineAuthentication-1114

Sample File	Sample Name	Panel	SQI	OS	SQ
29_E04_CellLineAuthentication1-10-1116.FSA	05	STR Profile 4-human		▲	▲

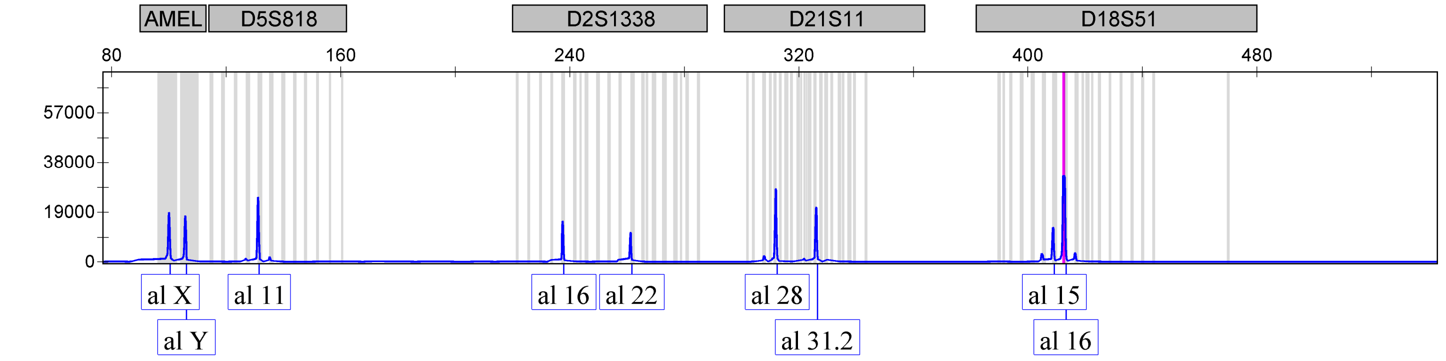

Sample File	Sample Name	Panel	SQI	OS	SQ
29_E04_CellLineAuthentication1-10-1116.FSA	05	STR Profile 4-human		▲	▲

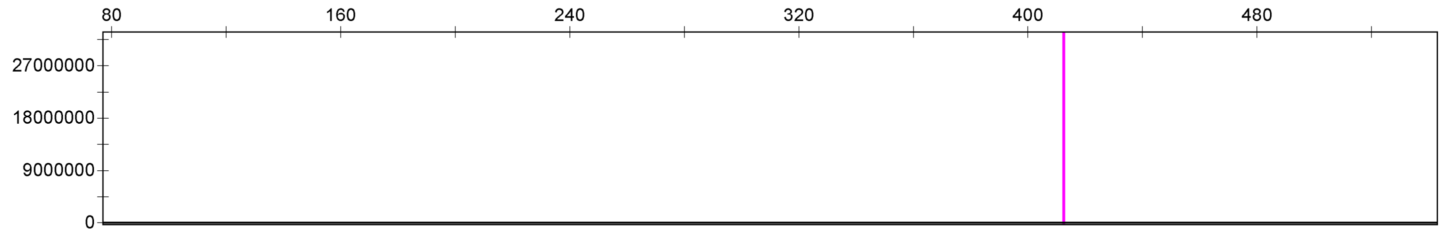

Supplement: Supplementary file 4 — C666-1 the certificate [file 41419_2019_1807_MOESM4_ESM.pdf]
