# Supplementary material for: Inhibition of ZIP4 reverses epithelial-to-mesenchymal transition and enhances the radiosensitivity in human nasopharyngeal carcinoma cells
Source: Cell Death Dis. 2019 Aug 5;10(8):588. doi: 10.1038/s41419-019-1807-7 (PMC6683154; doi:10.1038/s41419-019-1807-7)
Supplement: Supplementary file 5 — 293T Cell Line Authentication [file 41419_2019_1807_MOESM5_ESM.pdf]

Cell Line Authentication Service

Authentication STR Profiling Report

Sample from：Sichuan University

Sample Type：Cell Line

Testing Method：STR Genotyping

Report Time：2016-11-09

Shanghai Biowing Applied Biotechnology Co. Ltd Tel: +86-021-33559491 E-mail: biowing@vip.163.com
Address: Room 4F, 8 th Buiding,Guiguo Garden,NO.471 Guiping Road,Caohejing Development Zone,Shanghai

COMPANY STATEMENT

1. THIS REPORT IS ONLY RESPONSIBLE FOR THE SAMPLES ANALYZED.
2. THE TESTING RESULTS and THE ORGANIZATION NAME WILL NOT BE USED FOR ADVERTISEMENT, COMMERCIAL EXHIBITIONS, COMMERCIAL PERFORMANCE and OTHER COMMERCIAL ACTIVITIES.
3. OBJECTIONS SHOULD BE RAISED WITHIN FIFTEEN DAYS AFTER THE RECEIPT OF THIS REPORT.
4. THE PAPER REPORT WITH CONTENT ALTERING, ADDING OR WITHOUT THE STAMPED SEAL OF THE COMPANY ARE INVALID.

Shanghai Biowing Applied Biotechnology Co. Ltd Tel: +86-021-33559491 E-mail: biowing@vip.163.com
Address: Room 4F, 8 th Buiding,Guiguo Garden,NO.471 Guiping Road,Caohejing Development Zone,Shanghai

Sample Code

Table 1. Sample Code

Customer's code	Company Code
6	20161028-06

Sample Number:1

Sample Type: Cell line

Testing Type: STR

Sample From: Sichuan University

Testing Method:

DNA was extracted by a commercial kit from CORNING (AP-EMN-BL-GDNA-

250G). The ten STRs including one human locus were amplified by multiplex PCR and separated on ABI 3730XL Genetic Analyzer. The signals were then analyzed by the software GeneMapper.

Data Interpretation:

Cell lines were authenticated using Short T and em Repeat (STR) analysis as described in 2012 in ANSI St and ard (ASN-0002) by the ATCC St and ards Development Organization (SDO) and , mouse cell line authentication cytotechnology.2014;66:133-147.

Shanghai Biowing Applied Biotechnology Co. Ltd Tel: +86-021-33559491 E-mail: biowing@vip.163.com
Address: Room 4F, 8 th Buiding,Guiguo Garden,NO.471 Guiping Road,Caohejing Development Zone,Shanghai

Test Result

1. Result

Table 2. Matching information on the cell lines

Sample Code	Multi-allele	Cell line matched	Cross contamination	EV
20161028-06	Yes	293	DSMZ	0.944

- Multi-allele means some STR contain more than two loci.

2. Sample Description

A. 20161028-06: The STR results showed that have multiple alleles were found in this cell line, and no cross contamination of human cells was found in the cell line.

B. The DNA of the cell lines found to basic match the type of cell lines in a cell line retrieval, DSMZ database shows that cells called **293** . corresponding to the cell number **JCRB9068** .

Shanghai Biowing Applied Biotechnology Co. Ltd Tel: +86-021-33559491 E-mail: biowing@vip.163.com
Address: Room 4F, 8 th Buiding,Guiguo Garden,NO.471 Guiping Road,Caohejing Development Zone,Shanghai

3. Genotyping Result

STR and Amelogenin Genotyping Results of Cell line

Loci	Sample information			Cell Bank information			
	Sample name：20161028-06			Cell line name：293			
	Allele1	Allele2	Allele3	Allele1	Allele2	Allele3	Allele4
D5S818	8	10		8	9		
D13S317	12	12		12	12		
D7S820	11	12		11	12		
D16S539	9	13		9	13		
VWA	16	19		16	19		
TH01	7	9.3		7	9.3		
AMEL	X	X		X	X		
TPOX	11	11		11	11		
CSF1PO	11	12		11	12		
D12S391	19	20	21				
FGA	22	23					
D2S1338	19	20					
D21S11	28	29	30				
D18S51	18	19					
D8S1179	11	12	15				
D3S1358	15	16	17				
D6S1043	11	11					
PENTAE	7	15					
D19S433	15.3	16.3	17.3				
PENTAD	8.2	9.2					

Shanghai Biowing Applied Biotechnology Co. Ltd Tel: +86-021-33559491 E-mail: biowing@vip.163.com
Address: Room 4F, 8 th Buiding,Guiguo Garden,NO.471 Guiping Road,Caohejing Development Zone,Shanghai

Others

1. Genotyping Strategy and Site Distribution

Attached Table. Experimental Strategy and Sites

	Strategy 1	Strategy 2	Strategy 3	Strategy 4
1	TH01	TPOX	D3S1358	AMEL
2	D12S391	VWA	D13S317	D5S818
3	D7S820	D8S1179	D6S1043	D2S1338
4	CSF1PO	PENTAD	D16S539	D21S11
5	FGA		D19S433	D18S51
6	PENTAE			

- The allele match algorithm compares the 8 core loci plus amelogenin only, even though alleles from all loci will be reported when available.
- DSMZ tools was used to carry on the cell line comparison, which contains 2455 cell lines STR data from ATCC, DSMZ, JCRB ,ECACC，GNE and RIKEN databases. If the cell is not included in the above cell library, users need to compared with other databases.

2. Reference

1．Authentication testing of HEK 293T and HeLa cell lines have been performed by Shanghai Biowing Applied Biotechnology Co.,Ltd via STR profiling. STR profiles match the st and ards recommended for HEK 293T and HeLa cell lines authentication

2．AGS, NCI-N87, HGC-27 and HEK293 were STR-authenticated on Dec. 8, 2015 by Shanghai Biowing Applied Biotechnology Co. LTD, Shanghai, China

Technician: Jia nan Zhang

Check: Ning Qian

Person in Charge: Yang Bai

Issue date: 2016-11-09

Shanghai Biowing Applied Biotechnology Co. Ltd Tel: +86-021-33559491 E-mail: biowing@vip.163.com
Address: Room 4F, 8 th Buiding,Guiguo Garden,NO.471 Guiping Road,Caohejing Development Zone,Shanghai
