# Supplementary material for: Inhibition of ZIP4 reverses epithelial-to-mesenchymal transition and enhances the radiosensitivity in human nasopharyngeal carcinoma cells
Source: Cell Death Dis. 2019 Aug 5;10(8):588. doi: 10.1038/s41419-019-1807-7 (PMC6683154; doi:10.1038/s41419-019-1807-7)

Applied Biosystems
GeneMapper 4.0

Cell Line Authenticaion-5

Sample File	Sample Name	Panel	OS	SQ
06_F01_CellLineAuthentication1028-1102.fsa	6	STR Profile 1-dup		

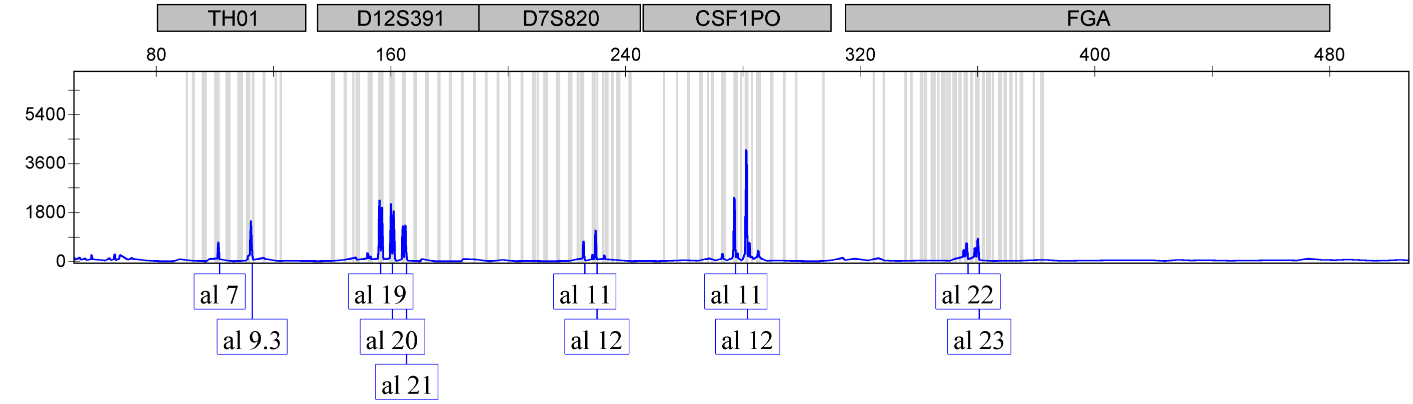

Sample File	Sample Name	Panel	OS	SQ
30_F04_CellLineAuthentication1028-1102.fsa	6	STR Profile 2-dup		

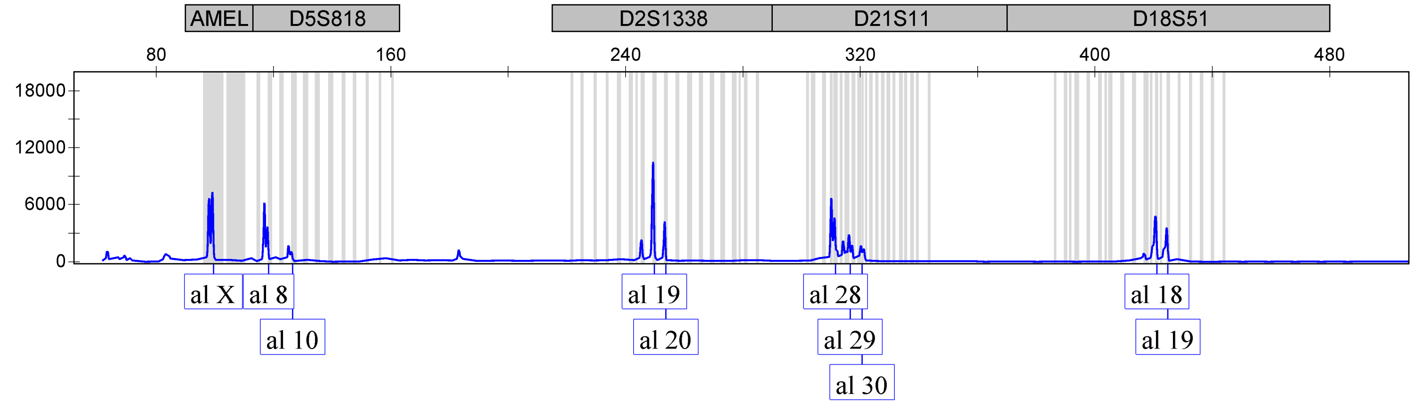

Sample File	Sample Name	Panel	OS	SQ
54_F07_CellLineAuthentication1028-1102.fsa	6	STR Profile 3-dup		

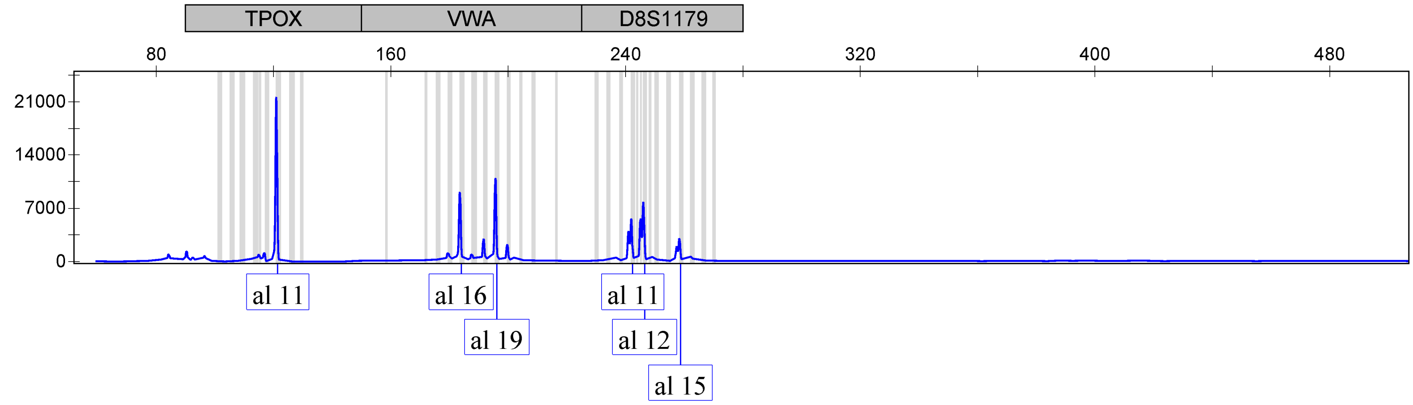

Applied Biosystems

GeneMapper 4.0

Cell Line Authenticaion-5

Sample File	Sample Name	Panel	OS	SQ
06_F01_CellLineAuthentication10284-1102.fsa	6	STR Profile 4-dup		

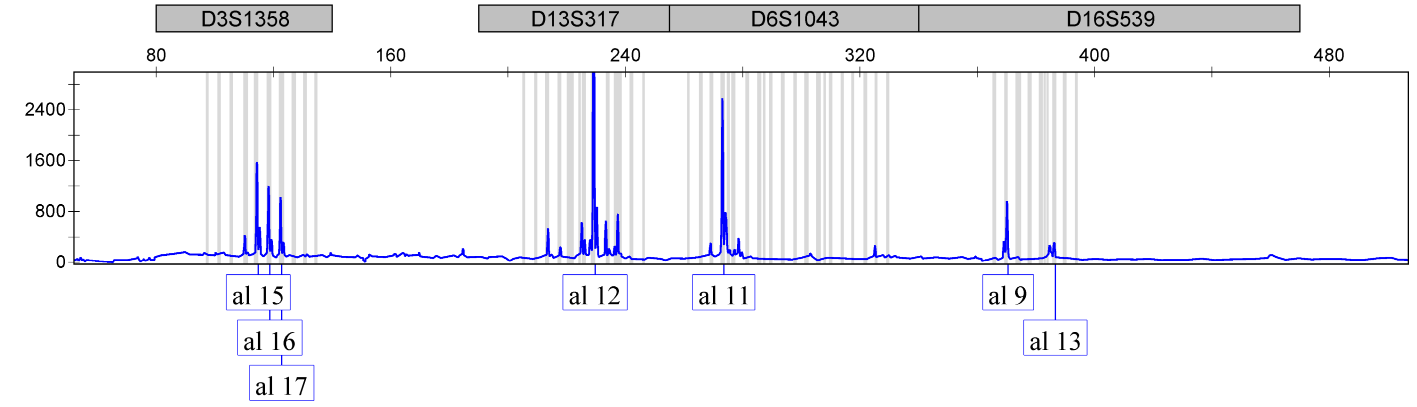

Sample File	Sample Name	Panel	OS	SQ
38_F05_CellLineAuthentication10284-1102.fsa	6	STR Profile 5-dup		

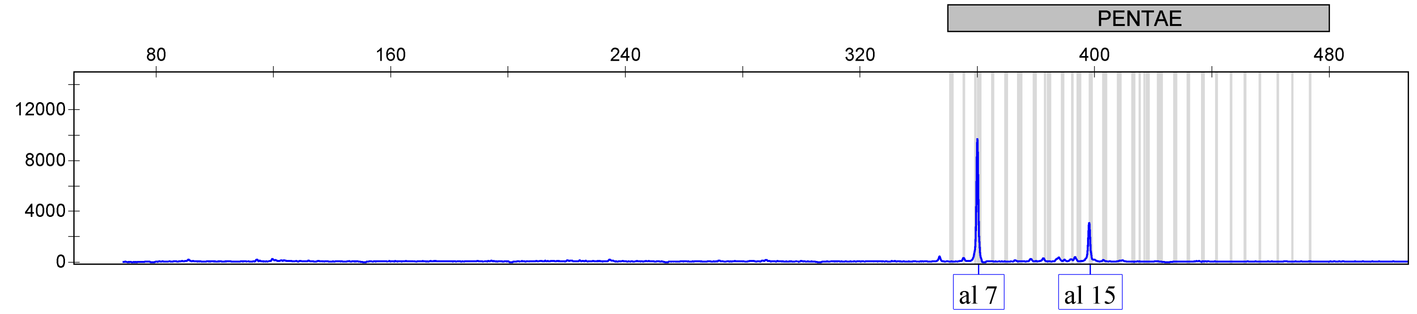

Sample File	Sample Name	Panel	OS	SQ
70_F09_CellLineAuthentication10284-1102.fsa	6	STR Profile 6-dup		

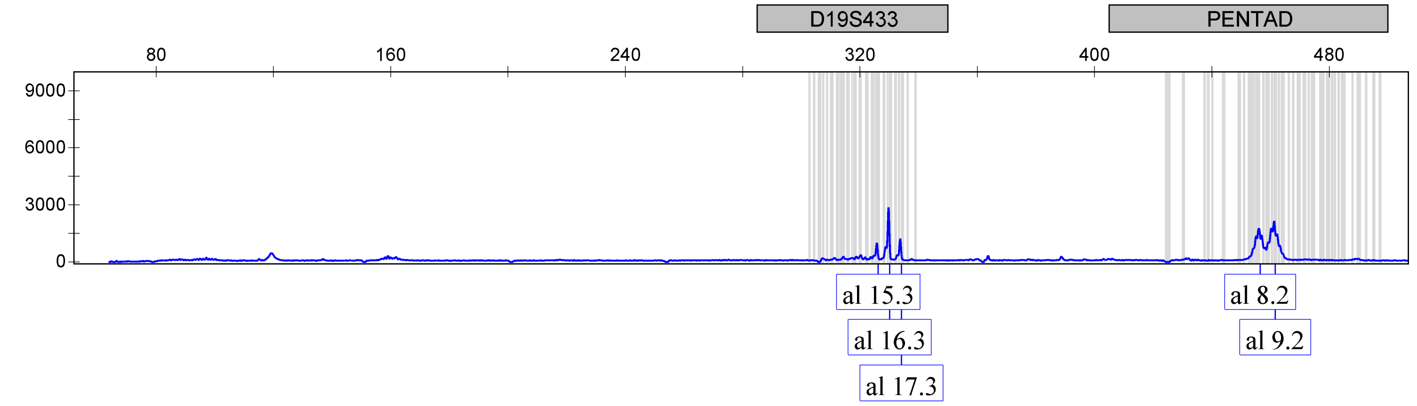

Supplement: Supplementary file 6 — 293T the certificate [file 41419_2019_1807_MOESM6_ESM.pdf]
